# Molecular Signature of Neuroinflammation Induced in Cytokine-Stimulated Human Cortical Spheroids

**DOI:** 10.3390/biomedicines10051025

**Published:** 2022-04-29

**Authors:** Kim M. A. De Kleijn, Kirsten R. Straasheijm, Wieteke A. Zuure, Gerard J. M. Martens

**Affiliations:** 1 Donders Centre for Neuroscience (DCN), Department of Molecular Animal Physiology, Faculty of Science, Donders Institute for Brain, Cognition and Behavior, Radboud University, 6525 GA Nijmegen, The Netherlands; wietekezuure@gmail.com (W.A.Z.); gerardus.martens@gmail.com (G.J.M.M.); 2 NeuroDrug Research Ltd., 6525 ED Nijmegen, The Netherlands; kirsten@neurodrugresearch.com

**Keywords:** brain, cortical spheroids, human, IL-1β, neuroinflammation, NFκB, RNA-sequencing, STAT, TNFα

## Abstract

Crucial in the pathogenesis of neurodegenerative diseases is the process of neuroinflammation that is often linked to the pro-inflammatory cytokines Tumor necrosis factor alpha (TNFα) and Interleukin-1beta (IL-1β). Human cortical spheroids (hCSs) constitute a valuable tool to study the molecular mechanisms underlying neurological diseases in a complex three-dimensional context. We recently designed a protocol to generate hCSs comprising all major brain cell types. Here we stimulate these hCSs for three time periods with TNFα and with IL-1β. Transcriptomic analysis reveals that the main process induced in the TNFα- as well as in the IL-1β-stimulated hCSs is neuroinflammation. Central in the neuroinflammatory response are endothelial cells, microglia and astrocytes, and dysregulated genes encoding cytokines, chemokines and their receptors, and downstream NFκB- and STAT-pathway components. Furthermore, we observe sets of neuroinflammation-related genes that are specifically modulated in the TNFα-stimulated and in the IL-1β-stimulated hCSs. Together, our results help to molecularly understand human neuroinflammation and thus a key mechanism of neurodegeneration.

## 1. Introduction

The pro-inflammatory cytokines tumor necrosis factor alpha (TNFα) and interleukin-1beta (IL-1β) play a crucial role in the homeostasis of the central nervous system (CNS), a number of neurological diseases, and CNS injury and repair [1,2,3,4,5,6,7]. For instance, levels of TNFα are elevated in patients suffering from Alzheimer’s disease (AD) [6], major depression [8,9], multiple sclerosis (MS) [10] and acute brain trauma and other encephalopathies [6]. Elevated IL-1β-levels have been reported in AD, Parkinson’s disease (PD), MS [11], epilepsy and stroke [3,7]. Furthermore, TNFα and IL-1β have important functions during human fetal brain development [12]. To explore how TNFα and IL-1β may contribute to disease onset, progression and maintenance is challenging, as studying the effects of these cytokines in an animal model entails translational issues and research with a cell line or co-culture cell model represents a reductionistic approach. Human brain organoids constitute an attractive three-dimensional (3D) model to study human brain developmental processes as well as the pathophysiological mechanisms underlying neurodevelopmental and neurodegenerative diseases, including neuroinflammatory processes [13,14,15,16].

Recently, we designed a protocol to generate human cortical spheroids (hCSs) comprising neuroectoderm-derived neural progenitor cells (NPCs), excitatory and inhibitory neurons, astrocytes and oligodendrocyte lineage cells as well as mesoderm-derived microglial and endothelial cells. In the current study, we set out to explore the effects of stimulating our hCSs for three time periods with TNFα and IL-1β. We use transcriptome-wide RNA-sequencing (RNA-seq) analysis to show that the major process induced by TNFα as well as by IL-1β is neuroinflammation. Central in this process are endothelial cells, microglia and astrocytes, and activation of the NFκB and STAT pathways. In addition to this equivalent impact of TNFα and IL-1β, we find that each of the two cytokines has specific and stimulation-time-dependent effects on hCS gene expression, and that IL-1β exhibits a faster self-inhibitory feedback response to dampen neuroinflammation than TNFα.

## 2. Materials and Methods

### 2.1. Culture of H9 Embryonic Stem Cells (ESCs)

H9 ESCs (WA09; WiCell, passage number 24) were grown feeder independently in E8 medium with Revitacell (diluted 1:100; Thermo Fisher, Waltham, MA, USA, A26445-01) supplemented with Penicillin-Streptomycin (Pen-Strep, 1:100, Thermo Fisher, 15140-122) on Corning Matrigel-coated surfaces (1:60; Corning, Wiesbaden, Germany, CLS354277) at 37 °C and 5% CO_2_. H9 ESCs were passaged twice per week at 1:20–25 splitting ratio. Cells were dissociated by a 5-min 37 °C incubation with Accutase (Invitrogen, Carlsbad, CA, USA, 00-4555-56), centrifuged at 1000 rpm for 5-min in Dulbecco’s phosphate-buffered saline (DPBS) and resuspended in E8 medium. Daily visual inspections were performed to ensure the undifferentiated state of the ESC cultures, and differentiated colonies were marked with a cell-culture marker and removed by micropipette suction. MycoAlert Mycoplasma tests (Lonza, Basel, Switzerland, LT07-118) were performed four times per year to ensure the mycoplasma-free status of the H9 ESC cultures.

### 2.2. Generation of hCSs

To generate hCSs, intact H9 ESC-colonies were detached with ReLESR (STEMcell technologies, Vancouver, BC, Canada, 05872) and low-adherence V-bottom 96-wells (S-Bio, #MS-9096VZ) were seeded with ~1.25 × 10^4^ cells per well. For the first seven days, spheroids were incubated with Spheroid Starter Medium (SSM: DMEM-F12 (Gibco, Madrid, Spain, 31331-028) with 20% knock-out serum (Gibco, 10828-028), 1% non-essential amino acids (Gibco, 11140050), 1% Pen-Strep and 0.1% 2-mercaptoethanol (Thermo Fisher, 31350-010) supplemented with 10 μM of the TGF-β signaling inhibitor SB-431542 (Sigma-Aldrich, Merck Life Science NV, Amsterdam, The Netherlands, S4317) and 1 μM of the BMP4 signaling inhibitor dorsomorphin (Sigma-Aldrich, P5499). Only during cell seeding was 2 μM ROCK inhibitor thiazovivin (Sigma-Aldrich, SML1045) added. The medium was then switched to Neurobasal-A spheroid medium (Thermo Fisher, 10888-022) with 2% B27 supplement minus vitamin A (Gibco; 12587-010), 1% GlutaMAX (Gibco, 35050-061) and 1% Pen-Strep (NSMnogel) (day 7 (d7) until day 25) and subsequently to NSMnogel medium with 15 μg/mL Geltrex (Thermo Fisher, A1413302) (NSM medium) until day 150 (d150). Between d14 and d25 half of the medium was refreshed daily. At d25, hCSs were transferred to 6-well low-attachment plates with a P1000 cut-open tip (~24 spheroids in 6 mL per dish). Between d25 and d150 half of the medium was refreshed every other day. Between d27 and d43 the NSM medium was supplemented with 20 ng/mL NT-3 (Sigma-Aldrich, SRP3128) and 20 ng/mL BDNF (Sigma-Aldrich, SRP3014). Between d43 and d51 the NSM medium was not supplemented with NT-3 and BDNF. Between d51 and d61 the NSM medium was supplemented with 10 ng/mL PDGF-AA (Sigma-Aldrich; H8291) and 10 ng/mL IGF1 (R&D systems, Minneapolis, MN, USA, 291-G1-200). From d61 until d73, half of the medium was refreshed with NSM medium containing 4 μM ketoconazole (Sigma-Aldrich, K1003). From d73 onwards, the medium consisted of NSM only. The sizes of our d150 hCSs were between 0.5 and 1.5 mm. During the entire growth period and all experiments, hCSs were kept at 37 °C and 5% CO_2_. Mycoplasma testing of the hCSs was performed four times per year.

### 2.3. Culture of Human Microglia (HMC3) and Human Oligodendrocyte (HOG) Cell Lines

HMC3 cells were purchased from ATCC (American Type Culture Collection, Manassas, VA, USA, CRL-3304). The HOG cell line was a kind gift of Dr. José Antonio López Guerrero (University of Madrid, Spain). The HMC3 and HOG cells were grown in EMEM (Lonza, BE12-662F) with 10% heat-treated FBS (Gibco, 10270106), 1% glutamax and 1% Pen-Strep, and passaged twice per week at a 1:20 splitting ratio (maximum passage number 35). The medium was refreshed every other day. Mycoplasma testing of the cell lines was performed tri-annually.

### 2.4. Pro-Inflammatory Cytokine Stimulation

For cytokine-stimulation experiments, d150 hCSs were transferred to low-attachment 6-well plates (Corning) that were placed on a gentle cell-culture incubator rocker in order to achieve an optimal flow of cytokines during the stimulation period. hCSs were incubated with 5 ng/mL TNFα (Sigma-Aldrich T0157-10UG; in PBS) or 5 ng/mL IL-1β (Sigma-Aldrich SRP3083-10UG; in PBS) for 4 h, 12 h or 36 h. The cytokine concentrations and stimulation time points were chosen based on our previous studies, studies utilizing 2D cell models [17,18,19,20,21], and the only study published thus far that dealt with cytokine (TNFα) stimulation of a 3D human brain organoid [22]. Following stimulation, spheroids were harvested for RNA isolation and the RNA was subjected to RNA-seq analysis as described below. Unstimulated hCSs from the same batch were used as controls. For pro-inflammatory stimulation experiments with HMC3 and HOG cells, the cells were incubated with 100 ng/mL LPS (Sigma-Aldrich, L4391-1MG) (dissolved in PBS) for 24 h (24 h) with subsequent incubation with 5 ng/mL TNFα (in PBS) and 5 ng/mL IL-1β (in PBS). Following stimulation, HMC3 and HOG cells were harvested for RNA isolation and the RNA was subjected to RNA-seq analysis, as described below.

### 2.5. RNA Isolation

Each hCS was transferred with a cut-open P1000 tip to 300 μL TRIzol reagent (Thermo Fisher; 15596026), snap frozen in liquid nitrogen and stored at −20 °C. For RNA isolation, hCSs were thawed and lysed in 400 μL TRIzol at room temperature for 5 min, then broken into smaller pieces with a P200 tip and incubated for an additional 30 min on ice. HMC3 and HOG cell pellets were snap frozen upon collection, thawed in 400 μL TriZOL and incubated for 30 min on ice. Chloroform was added to the lysed hCSs, HMC3 and HOG cells, and total RNA was extracted, precipitated with 100% 2-propanol and glycogen, washed twice with ice-cold 75% ethanol and dissolved in nuclease-free water. RNA was stored at −20 °C until further processing.

### 2.6. RNA-Seq Analysis

For RNA-seq analysis (BGI Genomics, Shenzhen, China), equal amounts of total RNA samples from 4–5 hCSs were pooled to a total of 700 ng RNA or 1.5 μg total RNA per HMC3 and HOG sample was used. For the determination of total RNA sample quality (RNA concentration, RNA integrity number (RIN) value, 28S/18S and the fragment length distribution), an Agilent 2100 Bioanalyzer (Agilent RNA 6000 Nano Kit, Agilent Technologies, Waldbronn, Germany) was used. Bulk RNA-seq analysis of total RNA samples (RIN value over 7.0, except for the hCSs LPS + TNFα + IL-1β sample: RIN 6.1) was performed on a DNBseq platform with a read depth of 30,000 reads; clean reads were mapped to reference genome h38 using HISAT2 (v2.0.4) and between 17,000 and 18,000 genes were identified. For the identification of differentially expressed genes (DEGs), clean reads were mapped to reference by Bowtie2 (v2.2.5), RNA expression levels were calculated with RSEM (v1.2.12) [23], and DEGs were detected with PoissonDis, as previously described [24] (fold change, FC, equal to or over 2.00 and false discovery rate, FDR, equal to or below 0.001). For the basal expression levels of all genes (fragments per kilobase of exon per million mapped fragments, FPKM), see Supplementary Information S1. For gene nomenclature, Log_2_ FC-values relative to 0 h stimulation and FDR-corrected *p*-values of the DEGs, see Supplementary Information S2. The RNA-seq data for the hCSs has been deposited under GEO accession number GSE200779, and for HMC3 and HOG under GEO accession number GSE200354. The RNA-seq data was validated by qPCR analysis and we calculated per time point the one-tailed Pearson correlation coefficient between the Log_2_ FC-values as obtained by RNA-seq and the mean Log_2_ FC-values as obtained by qPCR.

### 2.7. Bioinformatics Analysis

For an overview of the bioinformatics analysis of DEGs in the stimulated hCSs, see Supplementary Figure S1. The KEGG-pathway tool was used for pathway classification and functional enrichment (via the Phyper R-function) of DEGs in hCSs stimulated by TNFα or IL-1β for 4 h, 12 h or 36 h. The *p*-value per pathway was calculated via the hypergeometric distribution method, and the FDR was calculated per *p*-value (FDR < 0.01 are significantly enriched). The most biologically relevant pathways (core KEGG pathways) were identified based on a *p*-value below 10^−11^ and comprised “Cytokine-Cytokine receptor interaction”, “TNF signaling” and “NFκB signaling”.

Per stimulation time point, and for both TNFα or IL-1β, we constructed molecular landscapes reflecting the functional interactions between the proteins encoded by the DEGs in the three core KEGG pathways. We next constructed a molecular landscape based on the 95 DEGs that were present in the hCS DEG-lists of at least one TNFα and one IL-1β stimulation time point (a total of 1076 common genes) and that were annotated to one of the three core KEGG pathways.

The functions of the proteins encoded by the 95 DEGs were deduced on the basis of information obtained from GeneCards (Human Genome Database) and UniprotKB (Swiss Institute of Bioinformatics), and used to build the molecular landscape that was depicted in Biorender (www.Biorender.com; accessed on 22 March 2022).

To analyze the time courses of mRNA expression (hCS stimulation periods of 4 h, 12 h and 36 h relative to no stimulation), the expression profiles of the 1076 common DEGs were subjected to cluster analysis. Based on the Log_2_ transformed FC at the stimulation time points (4 h, 12 h or 36 h) relative to no stimulation and using a threshold of Log_2_ FC of 2.5 for the Boolean clustering strategy, this analysis revealed 22 clusters (Supplementary Table S1). As such, virtually all of the 1076 genes were allocated to a cluster (99.07% and 99.54% of the TNFα- and IL-1β-induced DEGs were clustered, respectively; Supplementary Table S2). A relatively low percentage of the DEGs overlapped between the TNFα- and IL-1β-clusters, except for clusters 8 and 22 (Supplementary Table S3); note that the two latter clusters contain a high number of DEGs (Supplementary Table S3) and that these DEGs display a relatively low Log_2_ FC at all three time points (between −2.5 and 0, or between 0 and 2.5) (Supplementary Table S1). The biological functions of the genes allocated to a TNFα-specific or IL-1β-specific cluster were deduced on the basis of information obtained from GeneCards and UniprotKB.

From a human fetal brain single-cell RNA-seq dataset (gestational weeks 22–23; [25]), we identified gene markers in five different brain regions (frontal cortex, parietal cortex, temporal cortex, occipital cortex and non-cortical regions) for neural progenitors (39–91 markers per brain region), excitatory neurons (691–1444 markers), inhibitory neurons (152–381 markers), astrocytes (92–262 markers), oligodendrocyte precursors (50–71 markers), microglia (491–708 markers) and endothelial cells (316–473 markers) on the basis of their mRNA expression levels in the other cell types being at least ten-fold lower (Supplementary Information S3). In order to estimate to which extent each brain cell type was affected by the cytokine treatment, for each cell type its number of marker genes present in the list of DEGs from our TNFα- or IL-1β-stimulated hCSs was determined, and this number was divided by the total number of marker genes for this cell type.

We further calculated the percentages of overlap between the DEGs in our TNFα- or IL-1β-stimulated hCSs and marker genes for neurodegenerative disease brain cell types as determined in [26] and derived from datasets of the superior frontal gyrus from AD (GEO accession number GSE48350; GSE26927), PD (GSE8397) and MS (GSE26927). In addition, we utilized the nCounter Analysis System from the Nanostring project (www.nanostring.com, accessed on 22 March 2022) to obtain genes genetically associated with AD, PD and MS, as well as genes annotated to the molecular signaling pathways “NFκB”, “neuroinflammation” and “inflammation” to determine their extent of overlap with the DEGs in our TNFα- or IL-1β-stimulated hCSs as well as their extent of overlap with the brain cell-type markers (derived from [25]) that were modulated by TNFα- or IL-1β.

### 2.8. Quantitative PCR (qPCR) Analysis

Following DNAse treatment of the RNA, cDNA synthesis was performed with the Revert Aid H-minus first-strand cDNA synthesis kit (Thermo Scientific). qPCR reactions were performed with SensiFAST™ Probe No-ROX (Meridian Bioscience-Bioline, Memphis, TN, USA, BIO-86005) on 1:10 diluted cDNA (for primer sequences, see Supplementary Table S4) in a Corbett Life Sciences Rotor-Gene 6000. We amplified cDNA for 50 cycles of 95 °C (5 s), 60 °C (10 s) and 72 °C (15 s), and melt-curve analysis was performed to confirm the correct amplicon size. Relative mRNA expression levels were calculated as described in Vandesompele et al. (2002) [27], on take-off (*ct*) values normalized to GAPDH/YWHAZ expression, with the following formula:

(1) $$\;Q_{x}=\mu_{eff}{}^{\left(ct_{min}-ct_{x}\right)}$$

In which $\mu_{eff}$ is the average amplification efficiency of all samples amplified with a certain primer pair, $ct_{min}$ is the minimum *ct* value of these samples and $ct_{x}$ is the *ct* value of the sample for which the *Q_x_*-value is calculated. This formula takes into account the mean amplification efficiency for one primer pair and scales all values between 0 and 1 for relative comparisons.

### 2.9. Immunocytochemistry

hCSs were fixed in 4% paraformaldehyde (PFA, prepared in phosphate buffer) for 45 min at room temperature, washed twice with PBS, incubated for 2 h in 15% sucrose in PBS (4 °C) and then incubated overnight in 30% sucrose in PBS (4 °C). Next, hCSs were embedded in O.C.T. Compound (VWR, Amsterdam, The Netherlands, 361603E) and cut into 5 μm sections on SuperFrost PLUS slides (VWR; J1800AMNZ) at −20 °C. For immunocytochemistry, sections were rehydrated for 5 min in PBS, blocked in home-made blocking buffer (5% goat/donkey/horse serum, 1% BSA, 1% Glycine, 0.1% D-lysine, in PBS) with or without 0.4% Triton-X100 for 30 min at room temperature. Antibodies were diluted in either PBS or PBS-Tween 20 0.1%. We used the antibodies MAP2 (Aves labs, Davis, CA, USA, AB_2313549; 1:200), TAU (Aves labs, AB_2313563; 1:200), GFAP (UC Davis/NIH NeuroMab facility, Davis, CA, USA, N206B/9; 1:500), O1 (Invitrogen, 14-6506-82; 1:200), P2RY12 (Biolegend, San Diego, CA, USA, 848002; 1:250), TMEM119 (Sigma, HPA051870; 1:500) and PECAM1/CD31 (NovusBio, Centennial, CO, USA, JC/70A; 1:100). All primary antibodies were incubated overnight at 4 °C and all secondary antibodies (1:500) for 2 h at room temperature. Washes were performed with PBS or PBS-Tween 20 0.1% (depending on the antibody protocol). DAPI was incubated in PBS (1:5000) for 30 min at room temperature. To test for any background staining, primary antibodies first validated in hCSs at a culture time point at which, based on mRNA expression data, no antigen expression was expected. Sections were imaged with the EVOS FL Auto 2 microscope (Thermo Fisher) with a 60× Olympus oil objective (Thermo Fisher).

### 2.10. Statistical Analysis

The difference between the mRNA expression levels of cell-type-specific markers in H9 hESCs and H9-derived d150 hCSs (Supplementary Figure S2A) was statistically tested with independent sample *t*-tests. When the *p*-value of the Levene’s test for equality of variances was below 0.05, we report the *p*-value without assumed equality of variances. To analyse the mRNA expression levels of a number of neuroinflammation-related genes modulated in stimulated hCSs (Supplementary Figure S3), One-way ANOVA with Dunnett’s post hoc test (one-sided) relative to the unstimulated (0 h) hCSs was used, which involved multiple testing correction. *p*-values below 0.05 were considered statistically significant. Errors bars in mRNA expression graphs represent the standard error of the mean.

## 3. Results

### 3.1. Neuroinflammation Is the Main Process Elicited in hCSs Stimulated with the Pro-inflammatory Cytokines TNFα and IL-1β

To investigate the effects of cytokine stimulation on our 3D hCSs comprising multiple brain cell types derived from both the neuroectoderm (neural progenitor cells, excitatory neurons, inhibitory neurons, astrocytes and oligodendrocytes) and the mesoderm (microglia and endothelial cells) (Supplementary Figure S2A,B), spheroids were incubated for either 4 h, 12 h or 36 h in the presence of 5 ng/mL TNFα or 5 ng/mL IL-1β. RNA-seq analysis of total RNA extracted from hCSs stimulated with TNFα and from control, unstimulated hCSs revealed 1119 DEGs (4 h TNFα; 646 upregulated, 473 downregulated), 1255 DEGs (12 h TNFα; 627 upregulated, 628 downregulated) and 1083 DEGs (36 h TNFα; 534 upregulated, 549 downregulated). In IL-1β-stimulated hCSs, we found 962 DEGs (4 h IL-1β; 420 upregulated, 542 downregulated), 588 DEGs (12 h IL-1β; 436 upregulated, 152 downregulated) and 602 DEGs (36 h IL-1β; 350 upregulated, 252 downregulated). The RNA-seq data was validated by qPCR analysis of a number of neuroinflammation-related genes modulated in the stimulated hCSs (Supplementary Figure S3), showing at all time points a highly significant correlation between the RNA-seq and qPCR data from the TNFα-stimulated hCSs (Supplementary Table S5) as well as between the RNA-seq and qPCR data from the IL-1β-stimulated hCSs (Supplementary Table S6).

**Figure 1:**
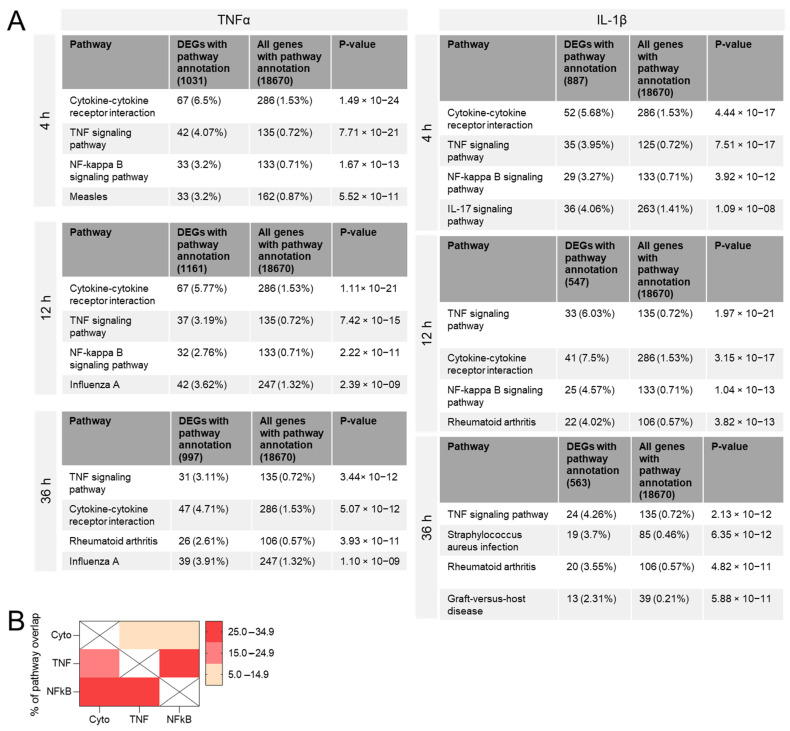
KEGG-pathway analysis of differentially expressed genes (DEGs) in human cortical spheroids stimulated with the pro-inflammatory cytokines TNFα or IL-1β for 4 h, 12 h, or 36 h. (**A**) In each case, the top-4 KEGG pathways (based on false discovery rate (FDR)--corrected *p*-value) are shown. On the basis of this pathway analysis and an FDR *p*-value cut-off of 10^−11^, we selected the three most frequent (core) KEGG pathways for further analysis, namely “Cytokine-cytokine receptor interaction” (Cyto; ko04060), “TNF signaling pathway” (TNF; ko04668) and “NF-kappa B signaling pathway” (NFκB; ko04065). (**B**) Degrees of overlap between the DEGs from the three selected core KEGG pathways.

KEGG-pathway analysis of the DEGs revealed that among the top-4 pathways at the three time points examined and in both TNFα- and IL-1β-stimulated hCSs, the three most frequently annotated (core) pathways were “Cytokine-cytokine receptor interaction” (ko04060), “TNF signaling pathway” (ko04668) and “NF-kappa B (NFκB) signaling pathway” (ko04065) (Figure 1A). The genes comprising the TNF signaling and NFκB signaling pathways showed the highest overlap (27.7% of the TNF signaling genes are also NFκB signaling genes), while the genes in the Cytokine-cytokine receptor interaction pathway displayed an overlap of only 7.8% and 9.5% with TNF signaling and NFκB signaling genes, respectively (Figure 1B). Gene ontology (GO) analysis of the DEGs also yielded mainly inflammation-related pathways, including “cytokine activity”, “cytokine receptor binding”, and “receptor ligand activity” (Supplementary Figure S4A). About half of the genes in the three core-KEGG-pathways were also present in the three core-GO-pathways (Supplementary Figure S4B). GO-analysis of the upregulated DEGs revealed mostly (neuro) inflammation-related pathways, while the downregulated DEGs were mostly related to “Extracellular matrix remodeling” (Supplementary Figure S4C).

### 3.2. Genes Dysregulated in TNFα- and IL-1β-Stimulated hCSs Are Involved in Positive and Negative Feedback Loops of Neuroinflammatory Signaling Pathways

To identify shared components of the neuroinflammatory response induced in the TNFα- as well as in the IL-1β-stimulated hCSs, we constructed a molecular landscape that showed the functional interactions between the proteins encoded by the common DEGs present in the three core KEGG pathways (Supplementary Figure S5A; Supplementary Information S4). The main biological processes apparent from the molecular landscape were NFκB- and STAT-dependent transcriptional activation. Crucially, the activation of transcriptional programs by NFκB (via *IKK, NFKBIA, NFKB1/2* and *RELB*) and STAT (via *JAK1/2, TYK2* and *STAT1/3/5/6*) (Supplementary Figure S5B,C) induces the expression of pro-inflammatory factors that on their turn trigger these programs to produce cytokines, chemokines and interleukins (such as *CXCL1/2/8*, *CCL9/20* and *IL1A/1B/5/6*), resulting in a positive feedback loop. Concomitantly, inhibitory NFκB-signaling genes (such as *TNFAIP3, BCL3* and *NFKBIA*) and inhibitory STAT-signaling genes (*SOCS3*) were rapidly induced following the cytokine stimulation (Supplementary Figure S5B,C), leading to a negative feedback loop. The presence of concurrent positive and negative NFκB- and STAT-feedback loops illustrates the complexity of the cytokine-induced neuroinflammatory response. These data show that at the three time points both TNFα and IL-1β elicit in hCSs an NFκB- and STAT-dependent regulation of various pro-inflammatory cytokines and chemokines, anti-inflammatory modulators, plasma membrane receptors as well as downstream transcription factors.

### 3.3. Stimulation-Time-Dependent Sets of DEGs in TNFα- and IL-1β-Stimulated hCSs

**Figure 2:**
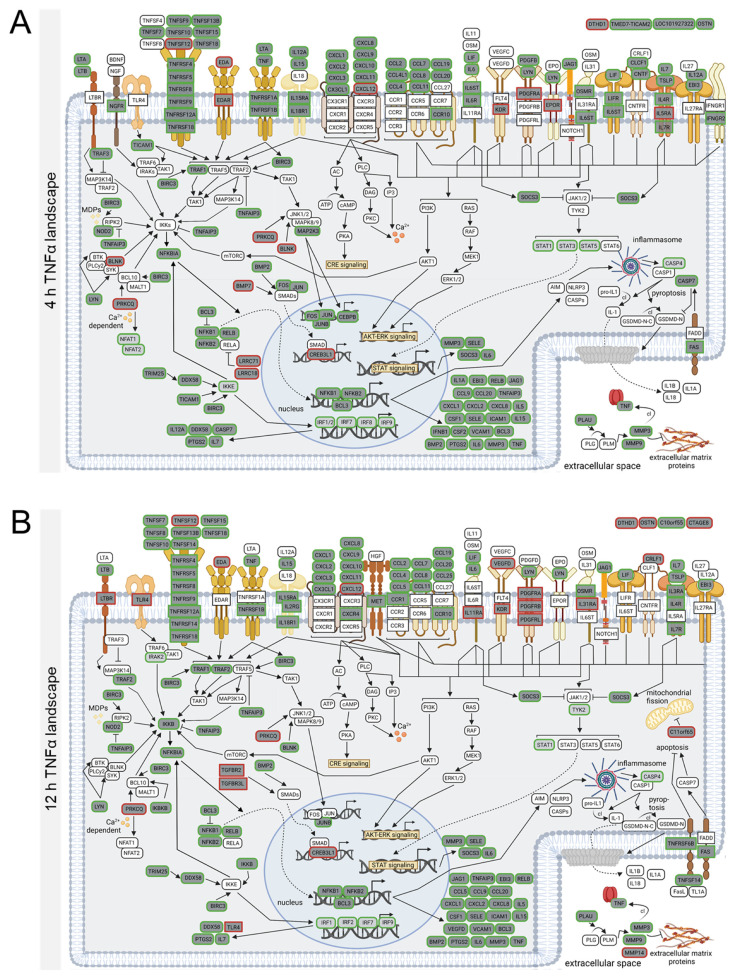

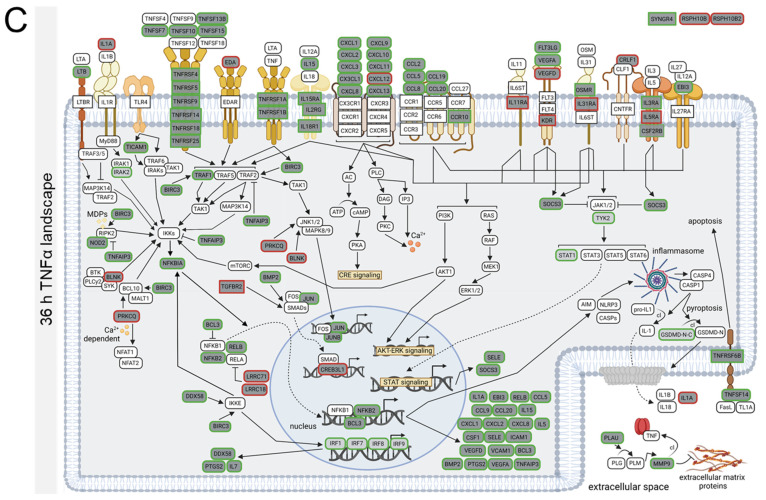
Molecular landscapes of the proteins encoded by the differentially expressed genes (DEGs) from the three core KEGG pathways (“Cytokine-cytokine receptor signaling”, “TNF signaling” and “NFκB signaling”) following TNFα stimulation of human cortical spheroids for (**A**) 4 h, (**B**) 12 h and (**C**) 36 h. Protein functions and interactions were deduced based on information obtained from UniprotKB (https://www.uniprot.org/; accessed on 1 January 2022) and Genecards (https://www.genecards.org; accessed on 15 January 2022). A detailed description of the protein-protein interactions occurring in these molecular landscapes can be found in Supplementary Information S5. Rectangularly framed protein: membrane-bound receptor; protein in green or red (rounded) rectangle frame: encoded by an up- or downregulated DEG, respectively; protein in dark-gray-filled (rounded) rectangle: encoded by a core KEGG pathway DEG; protein in light-gray-filled (rounded) rectangle: encoded by a DEG not in the core KEGG pathways; protein in white-filled (rounded) rectangle: not encoded by a DEG. Black arrows: stimulation or induction; inhibition arcs: inhibition; dotted arrows: translocation; fading arrow: enzymatic conversion; cl: cleavage.

**Figure 3:**
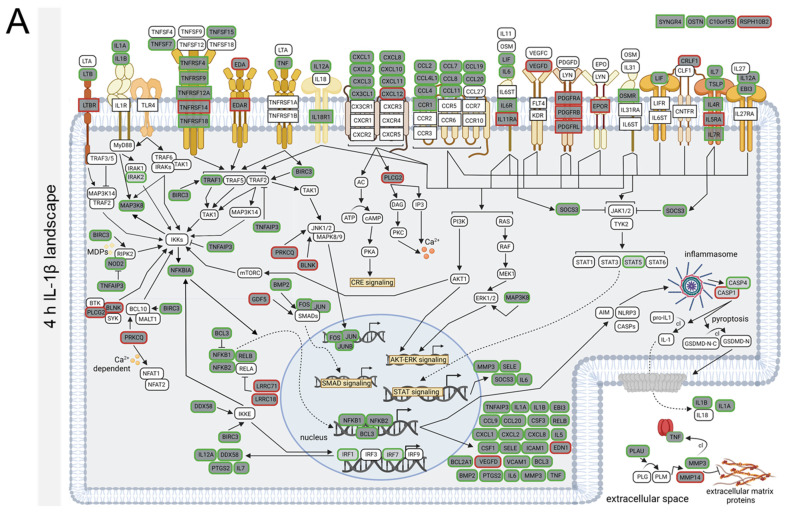

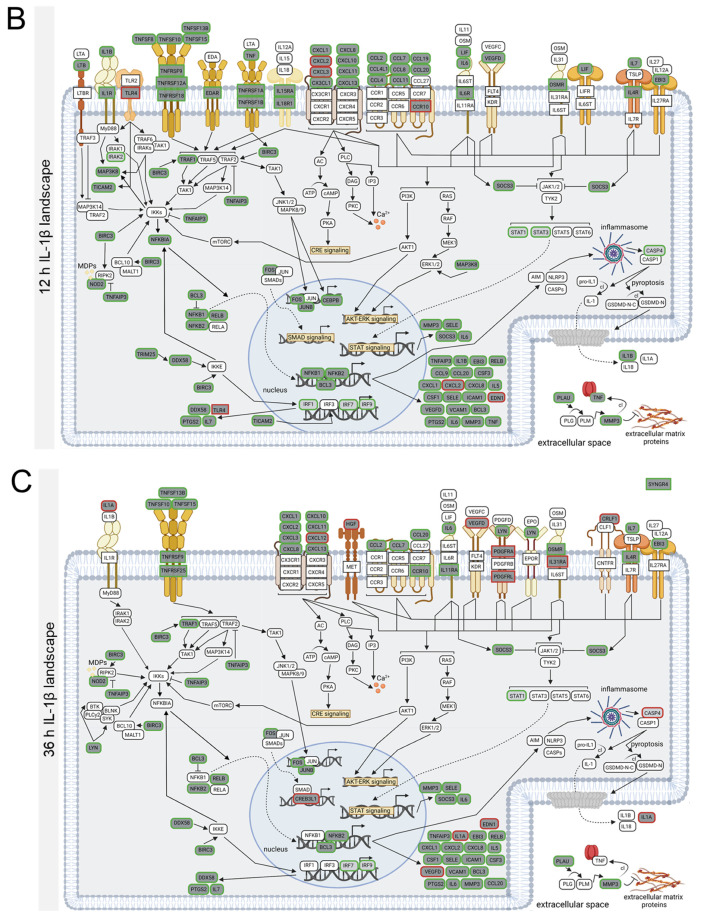
Molecular landscapes of the proteins encoded by the differentially expressed genes (DEGs) from the three core KEGG pathways (“Cytokine-cytokine receptor signaling”, “TNF signaling” and “NFκB signaling”) following IL-1β stimulation of human cortical spheroids for (**A**) 4 h, (**B**) 12 h and (**C**) 36 h. Protein functions and interactions were deduced based on information obtained from UniprotKB (https://www.uniprot.org/; accessed on 1 January 2022) and Genecards (https://www.genecards.org; accessed on 15 January 2022). A detailed description of the protein-protein interactions occurring in these molecular landscapes can be found in Supplementary Information S6. Rectangularly framed protein: membrane-bound receptor; protein in green or red (rounded) rectangle frame: encoded by an up- or downregulated DEG, respectively; protein in dark-gray-filled (rounded) rectangle: encoded by a core KEGG pathway DEG; protein in light-gray-filled (rounded) rectangle: encoded by a DEG not in the core KEGG pathways; protein in white-filled (rounded) rectangle: not encoded by a DEG. Black arrows: stimulation or induction; inhibition arcs: inhibition; dotted arrows: translocation; fading arrows: enzymatic conversion; cl: cleavage.

In the TNFα- and IL-1β-stimulated hCSs, we thus identified neuroinflammation-related pathways at all three time points. Yet, at the three time points the TNFα-modulated sets of DEGs showed an overlap of only ~50% (Supplementary Figure S6A), and the overlap between the three IL-1β-modulated DEG sets was only ~40% (Supplementary Figure S6B). Furthermore, the top-upregulated as well as the top-downregulated DEGs (based on Log_2_ FC) in the TNFα- and IL-1β-stimulated hCSs clearly showed different time-course expression profiles (Supplementary Figure S6C,D), indicating that for each of the two cytokines the compositions of the DEG sets were different at the three time points. On the basis of the TNFα-modulated DEGs annotated to the three core KEGG pathways, we constructed time-point-specific molecular landscapes that showed the marked differences in the various pathway compositions at the three time points (Figure 2A–C; Supplementary Information S5). Similarly, marked pathway composition differences were found by constructing time-point-specific molecular IL-1β landscapes (Figure 3A–C; Supplementary Information S6).

In the hCSs stimulated with TNFα for 4 h, we found the upregulated expression of TNF receptors *(TNFRs)4/5/8/9/12A/18*, IL receptors *(ILRs) 4/6/7/15/18/31* and chemokine receptor *CCR10*, and their ligands, such as *TNFSF7/9/10/13B/15/18*, *IL6/7/12A/15*, *CCL2/4/7/8/11/19/20*, and *CXCL1/2/3/8/9/10/11*, acting upstream of NFκB- and STAT-signaling (Figure 2A). The majority of these receptors and ligands remained upregulated following 12 h and 36 h of TNFα stimulation, except for *IL12A* at 12 h and *CCL4/7/11* and *TNFSF9/18* at 36 h (Figure 2B,C). In addition, the expression of the early response genes *FOS* and *JUN* was upregulated at 4 h TNFα (Figure 2A), and normalized at 12 h and 36 h (Figure 2B,C). Interestingly, already at the 4 h time point, the expression of NFκB-activating (*RIPK2*, *NFKB1*, *NFKB2*, *RELB*) as well as NFκB-inhibiting (*TNFAIP3*, *BCL3*) genes was upregulated (Figure 2A). A similar situation holds for 12 h and 36 h of TNFα stimulation (Figure 2B,C), except for *NFKB1* which was no longer a DEG at 36 h TNFα (Figure 2C). The expression of *TNF* itself was only induced following 4 h and 12 h of TNFα stimulation (Figure 2A,B), in line with a time-dependent normalization of the NFκB response. At all three TNFα-stimulation time points we observed a downregulation of the vascular endothelial receptor *KDR* (Figure 2A–C). Surprisingly, only at 12 h, but not at 4 h and 36 h, the upstream NFκB-inhibiting receptor *LTBR* and NFκB-stimulating receptor *TLR4* were downregulated (Figure 2B), indicating a simultaneous reduction in the activation and inhibition of the NFκB-pathway. In addition, only at 12 h were the SMAD-activating receptors *TGFBR2* and *TGFBR3L*, and the STAT-activating receptors *PDGFRB/L* downregulated (Figure 2B). Interestingly, at the 36 h time point we observed the downregulation of two genes related to cilia motility (*RSPH10B* and *RSPH10B2*) (Figure 2C).

Similar to the 4 h-TNFα-landscape, the molecular landscape of the 4 h IL-1β time point displayed upregulated expression of the NFκB-activating TNF-receptor superfamily members *TNFRSF4/9/12A/18*, but not *TNFRSF1A* and *TNFSFR1B* (Figure 3A), which showed upregulation only at 12 h IL-1β (Figure 3B). Remarkably, only 2, 3 and 4 out of the 7 TNFα-upregulated *TNFSF*s were induced at 4 h, 12 h and 36 h IL-1β, respectively (Figure 3A–C). In addition, at the 4 h time point the expression of various other receptors upstream of NFκB-signaling (*LTBR)* and STAT-signaling (*PDGFRB/L*, *IL11RA)* was already downregulated by IL-1β but not TNFα (Figure 2A and Figure 3A). A number of additional 4 h-TNFα-upregulated genes were already downregulated or not a DEG in the 4 h-IL-1β landscape, suggesting that the IL-1β-elicited neuroinflammatory response may have dampened faster than the TNFα-induced response. Indeed, at 12 h and 36 h following IL-1β stimulation *PDGFRB*, *CCR1* and *LTBR* expression was normalized to baseline levels and the 36 h IL-1β incubation resulted in further normalization to baseline levels of NFκB-activating genes such as *IL11RA, IL31RA, NFKB1, NFKBIA* and *CRLF1* (not DEGs anymore). Also in line with an early dampening of the NFκB-response and in contrast to the findings following 4 h, 12 h and 36 h TNFα stimulation (Figure 2A–C), the expression of the specific NFκB-inhibiting gene *PRKQC* was only downregulated at the 4 h IL-1β-stimulation time point and normalized to baseline levels at 12 h and 36 h IL-1β (Figure 3A–C). Interestingly, in contrast to the downregulation by 36 h TNFα we observed a downregulation of the cilium motility-related gene *RSPH10B* (but not *RSPH10B2*) only following 4 h of IL-1β-stimulation (Figure 3A). These results indicate that despite of the fact that the three core KEGG pathways modulated by TNFα and IL-1β at the three time points were the same, pronounced differences were found in the time-point-dependent and up- or downregulated sets of DEGs contained within these core pathways, whereby the IL-1β stimulation resulted in a faster activation and subsequent normalization of the NFκB- and STAT-pathways.

**Figure 4:**
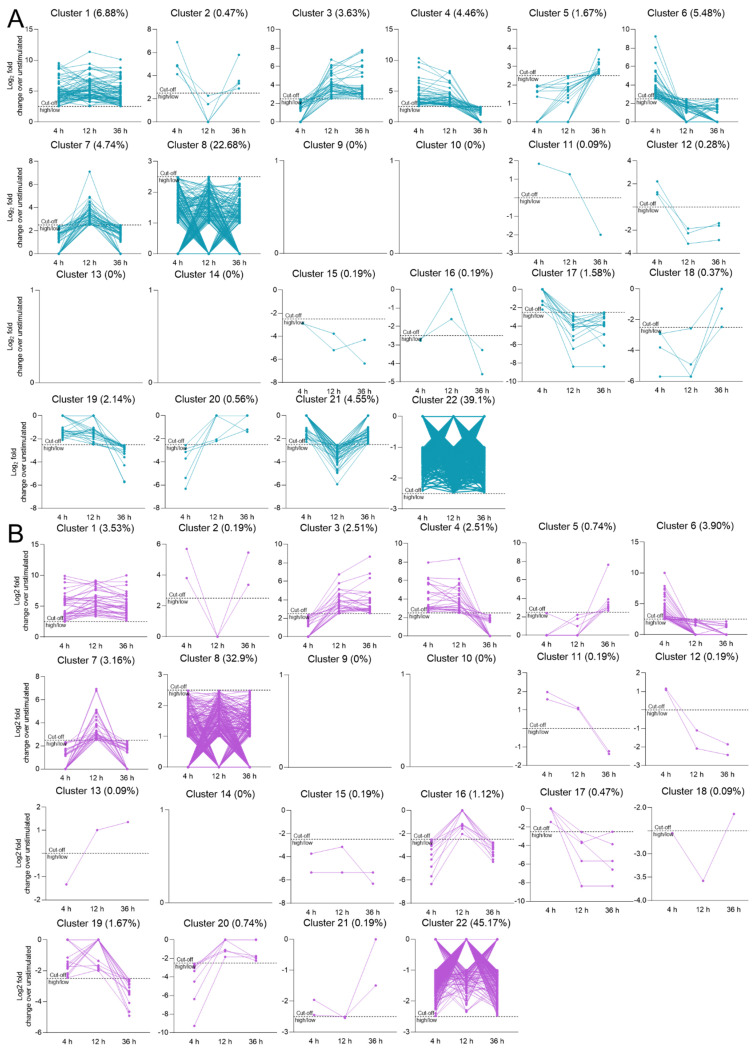
Cluster analysis of the expression profiles of the differentially expressed genes (DEGs) following (**A**) TNFα stimulation and (**B**) IL-1β stimulation of human cortical spheroids (hCSs) for 4 h, 12 h and 36 h. The 1076 DEGs overlapping between the TNFα- and IL-1β-modulated hCS DEG lists of the three stimulation time points were analysed.

Next, the 1076 DEGs overlapping between the two cytokine-modulated gene sets at the three stimulation time points were partitioned into 22 clusters, based on the time-course DEG expression profiles following TNFα-stimulation (Figure 4A) and IL-1β-stimulation (Figure 4B); Supplementary Tables S1 and S2 present the clustering strategy and the percentages of DEGs per cluster, respectively. For 12 out of the 22 clusters, we observed less than 25% overlap between the TNFα- and IL-1β-modulated DEGs (Supplementary Table S3; Supplementary Figure S7A), indicating that in the 12 clusters a significant number of genes showed a different time course of expression following either TNFα- or IL-1β-stimulation of the hCSs. To functionally characterize the DEGs with distinct TNFα- or IL-1β- induced time-course expression profiles within the 12 clusters, we performed a literature-based search for the biological roles of these genes. In seven out of the 12 clusters, a number of nonoverlapping genes displayed a notable functional similarity and a role related to neuroinflammation, such as antigen-presentation (4 out of 18 genes in cluster 5; 4/30 in cluster 3), TNF signaling (5/30 in cluster 3; 3/18 in cluster 5), interferon-signaling/NFκB-signaling/STAT-signaling (13/40 in cluster 4; 7/40 in cluster 7), chemoattraction and cell migration (6/42 in cluster 4), extracellular matrix (ECM; 8/49 in cluster 21), cytoskeleton-related (3/5 in cluster 17), proteasome (3/30 in cluster 3), Wnt signaling (3/30 in cluster 3) and fibrin/collagen-associated (9/54 in cluster 21) genes (Supplementary Figure S7B). This shows that beyond genes annotated to the three core KEGG pathways, other genes induced in the TNFα- and IL-1β-stimulated hCSs display distinct time-course expression profiles and are also related to neuroinflammation.

### 3.4. TNFα- and IL-1β-Specific Effects on Neuroinflammatory Signaling Pathways

**Figure 5:**
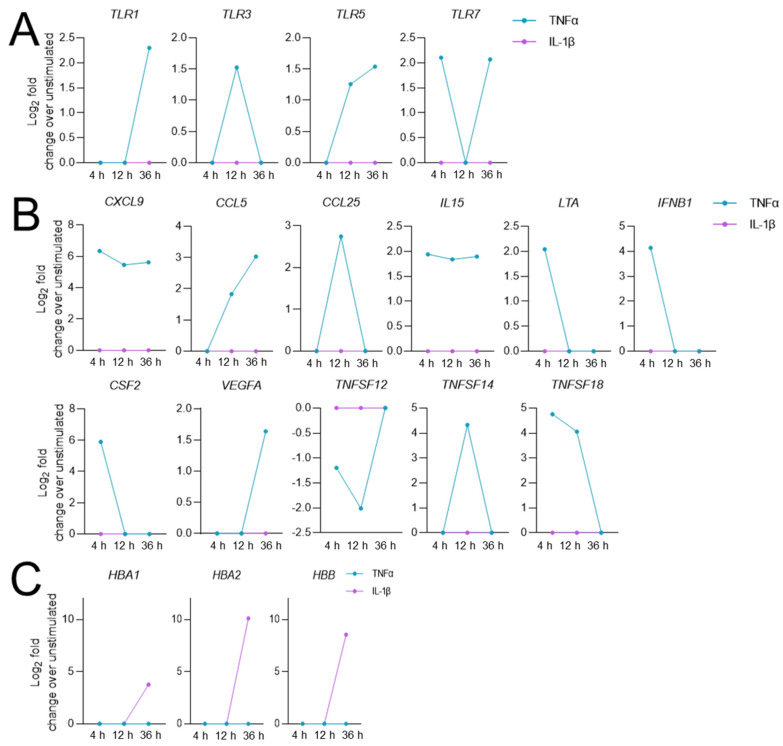
Gene expression profiles of (**A**) Toll-like receptor (TLR) subtypes, (**B**) Chemokines, cytokines and interleukins, and (**C**) Hemoglobins that are uniquely differentially expressed in human cortical spheroids following stimulation with either TNFα or IL-1β for 4 h, 12 h and 36 h.

In addition to the time-point-dependent differences between the expression profiles of the 1076 overlapping TNFα- and IL-1β-modulated DEGs (Supplementary Figure S8A), we also found unique expression profiles of DEGs induced by either TNFα- or IL-1β. Furthermore, the rather low number of genes that were present in the DEG lists of hCSs stimulated with TNFα- as well as in the IL-1β DEG lists of either of the three time points (e.g., 4 h: TNFα, 48% and IL-1β, 62%; 12 h: TNFα, 34% and IL-1β, 72%; 36 h: TNFα, 34% and IL-1β, 62%) (Supplementary Figure S8B,C) was also indicative of the existence of unique sets of TNFα-modulated DEGs and unique IL-1β-modulated DEG sets (from here on referred to as “category A” and “category B”, respectively; Supplementary Figure S8A). GO analysis revealed no significant pathways for the IL-1β-specific DEGs in category B (119 genes; Log_2_ FC >1.5 or <−1.5), while the GO molecular pathways “NAD+ nucleosidase activity” (7 DEGs) and “cytokine activity” (16 DEGs) were found for the TNFα-specific DEGs in category A (356 genes; Log_2_ FC >1.5 or <−1.5). Remarkably, 4 out of the 7 TNFα-specific DEGs from the “NAD+ nucleosidase activity” pathway were TLR subfamily members (*TLR1/3/5/7*) (Figure 5A), and 11 out of the 16 “cytokine activity” TNFα-specific DEGs represented chemokines, cytokines and other signaling molecules (*CXCL9*, *CCL5/25*, *IL15*, *LTA*, *IFNB1*, *CSF2*, *TNFSF12/14/18* and *VEGFA*) (Figure 5B). On the other hand, IL-1β but not TNFα induced the expression of three hemoglobin-related genes (*HBA1*, *HBA2*, *HBB*) (Figure 5C).

### 3.5. TNFα- and IL-1β-Induced Neuroinflammation Primarily Occurs in Endothelial, Microglia and Astrocyte Cell Populations, and Is More Related to MS Than to AD and PD

**Figure 6:**
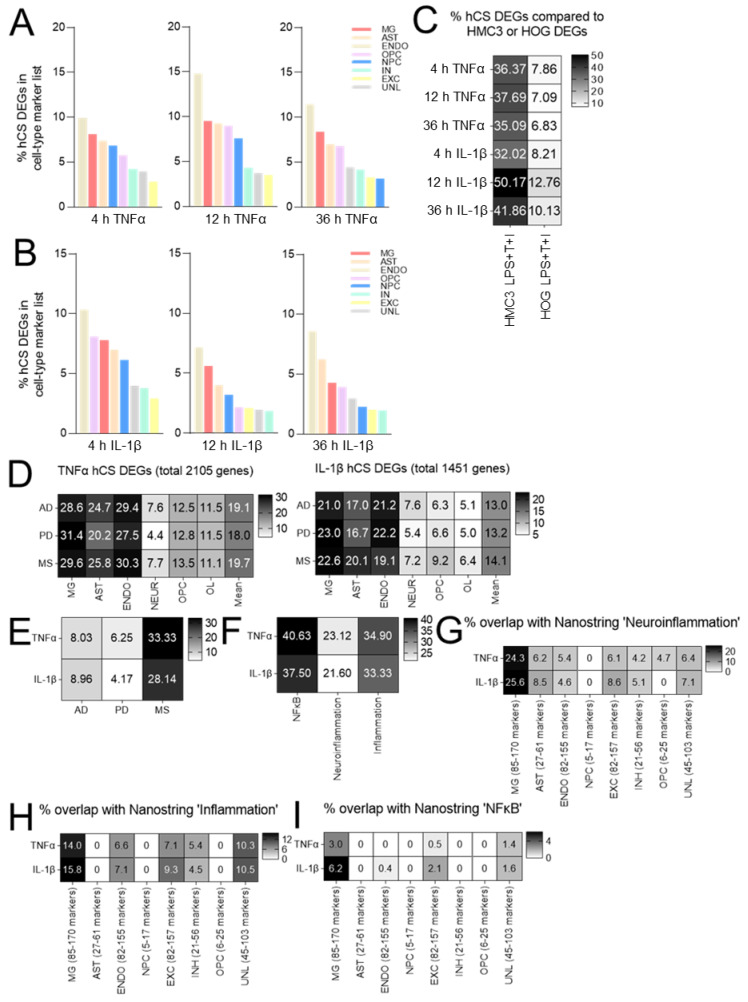
Differentially expressed genes (DEGs) in human cortical spheroids (hCSs) stimulated with TNFα or IL-1β are mainly expressed in endothelial cells, microglia and astrocytes. (**A**,**B**) Percentage of overlap between DEGs in hCSs stimulated for 4 h, 12 h and 36 h with (**A**) TNFα or (**B**) IL-1β and cell-type marker genes derived from [25], based on a cut-off of >10-fold expression difference with the other cell types; percentage of overlap was averaged over marker genes from five human brain regions. (**C**–**F**) Percentage of overlap between DEGs in hCSs stimulated for 4 h, 12 h and 36 h with TNFα or IL-1β and (**C**) DEGs in a human fetal microglia cell line (HMC3) or a human adult oligodendroglioma cell line (HOG) stimulated with pro-inflammatory factors (LPS for 24 h and subsequently TNFα and IL-1β for 24 h (LPS + T + I)), (**D**) cell-type marker genes for Alzheimer’s disease (AD), Parkinson’s disease (PD) and multiple sclerosis (MS) (cell-type marker genes derived from [26]), (**E**) genes associated with AD, PD and MS as derived from the Nanostring project, (**F**) NFκB-, neuroinflammation- and inflammation-associated genes as derived from the Nanostring project. (**G**) Percentage of overlap between DEGs in TNFα- or IL-1β-stimulated hCSs that are cell-type marker genes (derived from [25]), and neuroinflammation-associated genes, (**H**) inflammation-associated genes, or (**I**) NFκB-associated genes derived from the Nanostring project. MG: microglia; AST: astrocyte; ENDO: endothelial cell; OPC: oligodendrocyte precursor cell; NPC: neural progenitor cell; IN: inhibitory neuron; EXC: excitatory neuron; UNL: other, unlabeled cell types.

To gain insight into which of the cell types in our hCSs (neuroectoderm-derived neural progenitors, excitatory and inhibitory neurons, astrocytes and oligodendrocyte precursors, and mesoderm-derived microglia and endothelial cells) were primarily affected by the TNFα- and IL-1β-stimulations, we first defined cell-type-specific markers (Supplementary Information S3) based on a single-cell RNA-seq dataset of human fetal brain regions [25]. Using these brain cell-type-specific markers, we found that following the 4 h, 12 h and 36 h TNFα-stimulations in particular, the markers for endothelial cells and to a lesser extent microglia- and astrocyte-markers were the most prevalent in the lists of TNFα-modulated DEGs (Figure 6A). Endothelial-, microglia- and astrocyte-markers were also most prominent at all three time points in the IL-1β-modulated DEG lists, with, in addition, the clear presence of oligodendrocyte precursor cell (OPC) markers in the 4 h IL-1β-DEG list (Figure 6B). It is of further interest to note that the three sets of endothelial cell markers that were DEGs following 4 h, 12 h and 36 h of TNFα-stimulation overlapped for only ~50% (Supplementary Figure S9A). A similar situation holds for the degree of overlap among the microglia- and astrocyte-marker sets following the 4 h, 12 h and 36 h TNFα-stimulations, and the endothelial cell-, microglia- and astrocyte-marker sets following stimulation by 4 h, 12 h and 36 h of IL-1β (Supplementary Figure S9A,B). These results suggest that TNFα- and IL-1β-stimulation of hCSs affects glial and endothelial cell populations to a higher degree than the neuronal (progenitor) populations, and that the sets of cell-type-specific markers that are dysregulated by these cytokines vary among the three stimulation time points.

Interestingly, we found a considerable overlap between the DEG list from a microglial cell line (HMC3) that we stimulated with pro-inflammatory stimuli and the DEG lists from the 4 h-, 12 h- and 36 h TNFα-stimulated hCSs (35–37%) as well as between the HMC3 DEG list and the three IL-1β-stimulated hCS DEG lists (32–50%). In contrast, we only found an overlap of 6–12% between the DEG list from a pro-inflammatory stimuli-treated glial progenitor cell line (HOG) and the various DEG lists from the TNFα- and IL-1β-stimulated hCSs (Figure 6C). These findings are in line with the relatively high sensitivity of microglial populations to cytokines [28,29].

We next compared the lists of DEGs from the 4 h, 12 h and 36 h TNFα-stimulated hCSs as well as the 4 h, 12 h, 36 h IL-1β-stimulated hCS DEG lists with cell-type-specific marker genes identified on the basis of the suprafrontal gyrus microarray datasets derived from post-mortem brain tissues of AD-, PD- and MS-patients [26]. This analysis showed that at all three hCS stimulation time points the highest percentages of overlap were also found for microglia, endothelial cells and astrocytes; these percentages of overlap were roughly the same for the AD-, PD- and MS-datasets (Figure 6D). We then determined the percentages of overlap between the TNFα- and IL-1β-stimulated hCS DEG lists and genes genetically linked to AD, PD and MS (deduced from the Nanostring project). Intriguingly, our cytokine-modulated hCS DEGs are clearly more related to the genes associated with the neuroinflammatory disease MS (33% and 28% overlap with TNFα-stimulated and IL-1β-stimulated hCS DEGs, respectively) than with AD (8% and 9%, respectively) or with PD (6% and 4%, respectively) (Figure 6E). In addition, the TNFα- and IL-1β-modulated hCS DEGs showed a considerable degree of overlap with Nanostring-derived gene sets related to NFκB (~40%), inflammation (~35%) and neuroinflammation (~20%) (Figure 6F). Furthermore, a substantial percentage of the microglia-cell-type marker genes modulated in our hCSs by TNFα and IL-1β overlapped with genes from the Nanostring-derived neuroinflammation gene list (24.3% and 25.6%, respectively), much more than with the modulated cell-type marker genes of endothelial cells (5.4% and 4.6%), astrocytes (6.2% and 8.5%), NPCs (0% and 0%), excitatory neurons (6.1% and 8.6%), inhibitory neurons (4.2% and 5.1%), OPCs (4.7% and 0%) and other unlabeled cell populations (6.4% and 7.1%) (Figure 6G). Also, the overlap between the TNFα- and IL-1β-modulated microglia marker genes and the Nanostring-derived inflammation genes (14% and 15.8%, respectively) was higher than the overlap with other modulated cell-type marker genes (0–10.3% and 0–10.5%) (Figure 6H). Similarly, the overlap between the TNFα- and IL-1β-modulated microglia-cell-type marker genes and the Nanostring-derived NFκB gene list (3.0% and 6.2%, respectively) was clearly higher than the overlap with the other modulated cell-type markers (0–1.4% and 0–2.1%, respectively) (Figure 6I). These observations indicate that in the cytokine-stimulated hCSs most of the dysregulated (neuro)inflammation- and NFκB-associated genes are preferentially expressed in microglia.

## 4. Discussion

In this study, we performed unbiased RNA-seq and DEG analysis of TNFα- and IL-1β-stimulated hCSs containing multiple brain cell types (neuronal, astrocyte, oligodendrocyte, microglial and endothelial cell populations). The analysis revealed the induction of a neuroinflammatory response, with the NFκB- and STAT-pathways being the major pathways affected. This finding was found in both the TNFα- and IL-1β-stimulated hCSs, and at all three time points at which the spheroids were stimulated. However, the DEG compositions of these shared pathways were markedly different between the two cytokine-stimulated hCSs and among the three stimulation time points. In addition, a number of the common DEGs showed distinct time courses of expression. Furthermore, we identified TNFα- and IL-1β-specific DEGs at each of the three stimulation time points. The hCS cell types most responsive to TNFα- and IL-1β-stimulation, and thus driving the neuroinflammatory response, appeared to be endothelial cells, microglia and astrocytes.

As already at 4 h following TNFα- and IL-1β-stimulation the NFκB- and STAT-pathways were modulated, the induction of neuroinflammation represents an early event in the response of the hCSs. This is in agreement with the previously reported role of TNFα and IL-1β as early drivers of (neuro)inflammation [3,7,30,31], making these cytokines interesting targets for therapeutic interventions. Under pathophysiological conditions, the neuroinflammation-linked NFκB- and STAT-pathways are activated through the binding of extracellular ligands, including cytokines and chemokines, to plasma-membrane-bound receptors, such as the TNFR and ILR families, but also TLRs, various chemokine- and other cytokine receptors, pattern-recognition receptors and a number of G-protein-coupled receptors [32,33]. Expression of a variety of these ligands and receptors, such as *CXCL1/2/3/8/10/11/12*, *CCL2/4/7/8/19/20*, *TNFSF7/8/10/13B/15* and *TNFRSF4/9/14/18/25* as well as of the crucial transcriptional activation components of the downstream NFκB pathway (*NFKBIA*, *NFKB1*, *NFKB2*) was indeed affected in our cytokine-stimulated hCSs. In the canonical NFκB pathway, ligand-receptor binding activates multi-subunit IκB kinase (IKK) complexes that trigger the degradation of IκBa by phosphorylation [34,35], resulting in a rapid nuclear translocation of a number of NFκB family members, RelA and c-Rel. We indeed found upregulation of genes triggering this IKK-dependent IκBa-phosphorylation, such as *TRAF1*, *BIRC3*, *BLNK* and *LYN*. We further noticed that the receptors *PDGFR, ILR, CCR* and *CXCR*, and their ligands *PDGF, LYN, LIF, CCL2/4/4L1/7/8/11/19/20/25* and *CXCL1/2/3/8/9/10/11/12* that signal through STATs displayed affected expression in the cytokine-stimulated hCSs. In inflammatory and autoimmune diseases, the transcriptional activation of the STAT pathway is regulated via the transcription factors *STAT1/3/5/6* [36,37,38]. *STAT1* was upregulated at all TNFα time points and the 12 h- and 36 h-IL-1β time points. On the other hand, *STAT3/5* were only upregulated following 4 h of TNFα-stimulation, but not at later stimulation time points, while *STAT5* and *STAT3* were upregulated at 4 h and 12 h IL-1β-stimulation, respectively. These different time courses of *STAT1 versus STAT3/5* expression are in line with reports of antagonistic roles of *STAT1* (enhances inflammation) and *STAT3/5* (diminishes inflammation) in response to neuroinflammatory stimuli [39,40,41] and during ischemia [42].

Most (~80%) of the components of the NFκB- and STAT-pathways in the TNFα- and IL-1β-stimulated hCSs represented genes that were upregulated. In the downregulated gene set, we found a number of genes related to ECM remodeling, such as *COL4A4*, *COL6A* and *EMILIN3* following all three TNFα time points and *COL15A1*, *COL21A1*, *MFAP4* and *OGN* following all three IL-1β time points. Interestingly, a direct link between neuroinflammation and ECM remodeling/damage has been described during spinal cord injury [43], angiogenesis [44], and immune cell differentiation and migration [45], as well as microglial activation [46] and the pathogenesis of various neurological diseases [47,48,49], further indicating that our TNFα- and IL-1β- stimulated hCSs may well represent a valuable model of neuroinflammation.

A characteristic of the NFκB pathway is that the expression of cytokines activating NFκB is itself induced by this pathway, as such producing a positive feedback loop [34]. Transcription of the NFκB-activating cytokines *CCL9/20*, *CXCL1/2/8*, *IL1A/1B5/6/15* and *TNF*, regulated via the upregulation of NFκB-activating *TNFRs, TRAFs,* and *RIPK2*, was indeed increased in the stimulated hCSs. Interestingly, during inflammation, NFκB-activation also initiates negative feedback (self-inhibitory) loops that lead to the relief of inflammation [50,51]. In both the TNFα- and IL-1β-stimulated hCSs, we in fact found the downregulated expression of NFκB-activating factors, such as *BLNK*, and upregulated expression of NFκB-inhibiting factors, such as *BCL3*, *NFKBIA* and *TNFAIP3*. Most membrane-bound NFκB-activating receptors and downstream signaling molecules were upregulated following 4 h of TNFα-stimulation and therefore the hCSs were already in the NFκB-activation phase at this point in time. In addition, a number of inhibitory signaling genes were upregulated, reflecting an early self-inhibitory NFκB feedback loop. At the later TNFα-stimulation time points, the dampening of NFκB-activation was evident from the unaffected or downregulated expression of a number of NFκB-activating receptors and downstream molecules. Since in IL-1β-stimulated hCSs the dampening of the NFκB-pathway was already evident at 4 h, we conclude that the IL-1β-elicited neuroinflammatory response faded out earlier than the TNFα-induced response, which is in line with findings in a non-human, peripheral cell system (primary murine hepatocytes) [52].

An unbiased clustering strategy revealed differences in the time courses of the expression of the common TNFα- and IL-1β-dysregulated genes. For example, we identified clusters of genes displaying different TNFα- and IL-1β-modulated expression patterns and with roles in neuroinflammatory processes, such as chemoattraction (*ICAM4*, *ADGRG3)*, antigen-presentation (*HLA-DRB1*, *HLA-DPA1*, *HLA-C*, *B2M*, *HLA-DQA1*, *HLA-DMB*, *HLA-F*, *HLA-B*) and TNF signaling (*XAF1*); note that the bioinformatics analysis did not classify these genes within the three core KEGG pathways “Cytokine-Cytokine receptor signaling”, “TNF signaling” and “NFκB signaling”. Furthermore, we observed genes with distinct TNFα- and IL-1β-modulated time-course expression profiles and associated with structural/cytoskeletal functions, ECM remodeling, Wnt signaling and the proteasome, which have all been previously linked to neuroinflammation [48,53,54].

A remarkable finding was that the expression of four TLRs (*TLR1/3/5/7*), three specific cytokines (*CXCL9*, *CCL5/25)* and additional inflammatory molecules *(IL15*, *LTA*, *IFNB1*, *CSF2*, *VEGFA*, *TNFSF12/14/18)* was upregulated during the TNFα-, but not the IL-1β-induced neuroinflammatory response. At present, we do not know the explanation for this TNFα-specific effect. It is of further interest to note that 36 h of stimulation by IL-1β, but not TNFα, induced the expression of three hemoglobin genes (*HBA1*, *HBA2*, *HBB*). We speculate that IL-1β may activate a molecular cascade in endothelial cells that leads to the production of these hemoglobins, which are modulators of vascular tone [55,56]. This is in line with the fact that the IL-1R is highly expressed in endothelial cells and involved in angiogenic responses [57,58,59], although TNFα receptors also exhibit endothelial-cell expression and angiogenic activity [60,61]. Specific TNFα- and IL-1β effects have been previously reported in peripheral immune cells and brain cells, namely that the two cytokines differentially affect the expression of certain cytokines (e.g., CXCL8/IL8; [62]), pro-adhesion molecules (e.g., ICAM1 and VCAM1; [63]), and pentraxin 3 [64]. Thus, despite the fact that both TNFα and IL-1β have a pro-inflammatory function, the molecular neuroinflammatory signatures elicited by these cytokines appear to be markedly different.

NFκB orchestrates the neuroinflammatory response to ATP, ion imbalance, excess glutamate, oxidative stress and cytokines in CNS cells, such as microglia, astrocytes, neurons and oligodendrocytes [35,57,65,66]. We found that the neuroinflammatory response elicited by TNFα and IL-1β in hCSs is primarily driven by endothelial cells, microglia and astrocytes, consistent with previous findings showing that under inflammatory conditions the two cytokines affect (micro)glial functions [67,68] and angiogenesis [63,69,70]. In view of the pivotal support functions of endothelial cells, microglia and astrocytes in the brain [71,72,73], activation of these cells in the hCSs may on its turn have affected other brain cell types such as oligodendrocytes and neuronal (progenitor) populations. Such aberrant neuroinflammation-provoked crosstalk between glial cells and other brain cell types has been previously observed in neuroinflammatory diseases [74,75,76]. We found that the list of DEGs from our TNFα- and IL-1β-stimulated hCSs showed a higher similarity to that from pro-inflammatory-stimulated human fetal microglia (HMC3) than human adult glial progenitor (HOG) cells, indicating that these cell lines do recapitulate the known difference in sensitivity of the various brain cell types to cytokines [28,29,77]. Nevertheless, one has to realize that, in vitro, in the absence of other cell types and in a 2D environment, TNFα- or IL-1β-stimulated brain cell lines may well respond markedly different from our 3D TNFα- and IL-1β-stimulated hCSs.

A prolonged activation of NFκB in brain cell types appears to contribute to pathogenic neurological processes, [78] and NFκB-associated neuroinflammation is a hallmark of various neurological diseases, including AD, PD and MS [35,79]. Our comparison of the lists of TNFα- and IL-1β-modulated hCS DEGs with genes genetically associated with AD, PD or MS revealed that the highest percentage of DEGs was related to MS, in line with the clear neuroinflammatory contribution to the pathogenesis of this disease [80,81]. Interestingly, when comparing the cytokine-modulated hCS DEG lists with the transcriptomes of various AD-, PD- and MS-cell types, we did not find a preference for MS-related genes. We presume that this apparent discrepancy is explained by the fact that the genetically linked genes contribute to the development of the disease, while the transcriptomes reflect endpoints of disease pathogenesis.

Besides its relevance for the pathogenesis of a number of neurological diseases, more knowledge about NFκB-mediated neuroinflammation is crucial, since this complex process is associated with a variety of opposing responses, from cell survival to cell death [79]. Our molecular dissection of the TNFα- and IL-1β-induced neuroinflammatory responses was performed in ESC-derived self-assembling 3D-hCSs containing all major brain cell types. Therefore, the processes elicited in our cytokine-stimulated hCSs may well mimic the neuroinflammatory response in the brains of neurological disease patients, in particular during MS pathogenesis, and the identification of their molecular signatures contributes to the development of TNFα-, IL-1β- and NFκB-directed therapies.

## 5. Patents

The protocol to generate human cortical spheroids presented here is part of a pending patent application.

## Data Availability

The raw RNA-seq data for the hCSs has been deposited under GEO accession number GSE200779 (and are also available in Supplementary Information S1 and S2), and for HMC3 and HOG under GEO accession number GSE200354.

## Supplementary Materials

The following supporting information can be downloaded at: https://www.mdpi.com/article/10.3390/biomedicines10051025/s1 [82,83,84,85,86,87,88,89,90,91,92,93,94,95,96,97,98,99,100,101,102,103,104,105,106,107,108,109,110,111,112,113,114,115,116,117,118,119,120,121,122,123,124,125,126,127,128,129,130,131,132,133,134,135,136,137,138,139,140,141,142,143,144,145,146,147,148,149,150,151,152,153,154,155,156,157,158,159,160,161,162,163,164,165,166,167,168,169,170,171,172,173,174,175,176,177,178,179,180,181,182,183,184,185,186,187,188,189,190,191,192,193,194,195,196,197,198,199,200,201,202,203,204,205,206,207,208,209,210,211,212,213,214,215,216,217,218,219,220,221,222,223,224,225,226,227,228,229,230,231,232,233,234,235,236,237,238,239,240,241,242,243,244,245,246,247,248,249,250,251,252,253,254,255,256,257,258,259,260,261,262,263,264], Figure S1: Overview of the analysis of differentially expressed genes (DEGs) in human cortical spheroids (hCSs) stimulated with the pro-inflammatory cytokines TNFα or IL-1β for 4 h, 12 h and 36 h. Per time point, a molecular landscape for TNFα-stimulated or IL-1β-stimulated hCSs was built based on functional interactions between the proteins encoded by the DEGs in the three core annotated KEGG pathways. A further molecular landscape was constructed based on the DEGs that were common in the three core annotated KEGG pathways of the hCSs stimulated with TNFα and IL1β for 4 h, 12 h and 36 h. Clustering analysis of the common 1076 DEGs was performed to reveal 22 clusters of time-course gene expression profiles. Also, DEGs unique in TNFα- or IL-1β-stimulated hCSs were grouped into a distinct category (category A for TNFα and category B for IL-1β). Biological functions were deduced based on information obtained from UniprotKB (https://www.uniprot.org/; accessed on 1 January 2022) and Genecards (https://www.genecards.org; accessed on 15 January 2022); Figure S2: Our human cortical spheroids (hCSs) contain neuroectoderm- and mesoderm-derived cell types. (A) Levels of mRNA expression (measured by qPCR) of neural progenitor marker PAX6, mature neuron marker NEFL, excitatory neuron marker SLC17A7, inhibitory neuron marker GAD1, astrocyte marker AQP4, oligodendrocyte marker MBP, microglia marker CX3CR1 and endothelial cell marker SPARC in embryonic stem cell line H9 (n = 3) and in hCSs (n = 3) at day 150 (d150), relative to embryonic H9 stem cells. *** *p* < 0.001, ** *p* < 0.01, * *p* < 0.05, # *p* < 0.1. (B) Representative immunocytochemistry images of d150 hCS stainings for neuronal dendritic marker MAP2 and axonal marker TAU, astrocyte marker GFAP, oligodendrocyte marker O1, microglia markers P2RY12 and TMEM119, and endothelial surface marker PECAM1; Figure S3: Pro-inflammatory stimuli increase mRNA expression of neuroinflammatory-related genes in human cortical spheroids (hCSs). Normalized mRNA expression levels of IL-6, CXCL8, CCL5, CCL20, NFKB1, STAT1 and STAT3 at (A) various TNFα (5 ng/ml) incubation time points and (B) various IL-1β (5 ng/ml) incubation time points and quantified by qPCR. *** *p* < 0.001, ** *p* < 0.01, * *p* < 0.05, # *p* < 0.1; Figure S4: Gene ontology (GO) analysis of differentially expressed genes (DEGs) in human cortical spheroids stimulated with TNFα or IL-1β for 4 h, 12 h and 36 h. (A) GO top-4 pathways (based on false discovery rate (FDR)-corrected *p*-value). (B) Number of DEGs in the three core KEGG pathways (left), the three most-often found GO pathways (right) and overlapping between the three core KEGG and GO pathways (214 out of 436 (49.1%) KEGG genes are also GO genes; 214 out of 604 (35.4%) GO genes are also KEGG genes). (C) GO top-4 pathways (based on FDR *p*-value) resulting from the analysis of upregulated DEGs (UP) or downregulated DEGs (DOWN); Figure S5: (A) Molecular landscape constructed on the basis of differentially expressed genes (DEGs) in human cortical spheroids following stimulation with TNFα and with IL-1β for 4 h, 12 h and 36 h (“common” DEGs). Protein functions and interactions were deduced based on information obtained from UniprotKB (https://www.uniprot.org/; accessed on 1 January 2022) and Genecards (https://www.genecards.org; accessed on 15 January 2022). A detailed description of the protein-protein interactions occurring in this molecular landscape can be found in Supplementary Information S4. Rectangularly framed protein: membrane-bound receptor; protein in dark-gray-filled (rounded) rectangle: encoded by a core KEGG pathway DEG; protein in light-gray-filled (rounded) rectangle: encoded by a DEG not in the core KEGG pathways; protein in white-filled (rounded) rectangle: not encoded by a DEG. Black arrows: stimulation or induction; inhibition arcs: inhibition; dotted arrows: translocation; fading arrows: enzymatic conversion; cl: cleavage. Encircled numbers refer to the two main dysregulated molecular pathways; 1: NFκB signaling, 2: STAT-dependent transcription. (B) Gene expression profiles of NFκB-master regulator genes NFKBIA, NFKB1, NFKB2, RELB, BCL3 and TNFAIP3. (C) Gene expression profiles of STAT-master regulator genes STAT1, STAT3 and SOCS3.; Figure S6: Number and time-course expression profiles of upregulated (left) and downregulated (right) differentially expressed genes (DEGs) in human cortical spheroids stimulated with TNFα (A and C) or IL-1β (B and D) for 4 h, 12 h and 36 h. For time-course expression profiles, per time point top DEGs are based on Log2 fold change with a cut-off of 4 (upregulated DEGs; UP) or -4 (downregulated DEGs; DOWN); Figure S7: Cluster analysis and biological functions of common differentially expressed genes (DEGs) in human cortical spheroids stimulated with TNFα and IL-1β for 4 h, 12 h or 36 h. (A) Percentages of common DEGs in TNFα- and IL-1β-clusters resulting from DEG cluster analysis. Dotted red line: cut-off (overlap score between corresponding TNFα- and IL-1β-clusters <25%), (B) Biological functions of cluster DEGs with a <25% overlap score between corresponding TNFα- and IL-1β-clusters. Functions were deduced from literature-based studies; Figure S8: Number of differentially expressed genes (DEGs) in human cortical spheroids stimulated with TNFα or IL-1β for 4 h, 12 h and 36 h. (A) Number of common DEGs dysregulated at one of the three time-points of TNFα- and IL-1β-stimulation (overlap), and number of DEGs uniquely dysregulated following TNFα-stimulation (left) and IL-1β-stimulation (right). (B) Number of common DEGs overlapping between the TNFα- and IL-1β-DEG lists (overlap), and number of DEGs unique to the TNFα-DEG list (left) and IL-1β-DEG list (right) per stimulation time point. (C) Number of common DEGs overlapping between the TNFα- and IL-1β-DEG lists (overlap), and number of DEGs unique to the TNFα-DEG lists (left) and IL-1β-DEG lists (right) among the various stimulation time points; Figure S9: Number of endothelial cell-, microglia- and astrocyte-marker genes that are dysregulated in human cortical spheroids following stimulation with (A) TNFα or (B) IL-1β for 4 h, 12 h or 36 h. Marker genes (Supplementary Information S3) were based on a single-cell RNA-seq dataset of human fetal brain regions [25]; Table S1: Boolean clustering of the time-course expression profiles of common differentially expressed genes (DEGs) selected on the basis of their presence in the DEG-lists of human cortical spheroids (hCSs) stimulated with TNFα for 4 h, 12 h or 36 h as well as in the DEG-lists of hCSs stimulated with IL-1β for 4 h, 12 h or 36 h. In the column “Pattern”, “high” refers to a Log2 fold change (FC) >2.5 or <−2.5 and “low” refers to a Log2 FC between 0 and 2.5 or between −2.5 and 0. In the column “Direction”, “pos” indicates that the FC-value is above 0 and “neg” indicates that the FC-value is below 0. The boundary at the DEG threshold of Log2 FC = 1 could not be set as some genes were only a DEG at one time point and thus values with a Log2 FC between 1 and −1 were also present in the dataset; Table S2: Listed are per cluster (see Figure 4) the percentages of TNFα-modulated differentially expressed genes (DEGs) and the percentages of IL-1β-modulated DEGs. Percentages are relative to the total number of common TNFα- and IL-1β-modulated DEGs (1076 DEGs); Table S3. Listed are per cluster (see Figure 4) the number of genes in the time-course expression cluster of TNFα- and IL-1β-modulated differentially expressed genes (DEGs), the number of genes common in the time-course expression clusters of TNFα- and IL-1β-modulated DEGs and the percentage of genes common in the time-course expression clusters of TNFα- and IL-1β-modulated DEGs; Table S4: Primer sequences used for qPCR validation of RNA-seq data in day-150 human cortical spheroids; Table S5: Validation of RNA-sequencing (RNA-seq) data by quantitative PCR (qPCR) analysis of the expression of genes modulated in human cortical spheroids (hCSs) stimulated with TNFα for 4 h, 12 h or 36 h. Listed are Log2 fold-change (FC) values in the stimulated hCSs over the unstimulated (0 h) hCSs. Correlation RNAseq-qPCR 4 h: 0.685, *p*-value: 0.001; correlation RNAseq-qPCR 12 h: 0.862, *p*-value: <0.001; correlation RNAseq-qPCR 36 h: 0.819, *p*-value: <0.001; Table S6: Validation of RNA-sequencing (RNA-seq) data by quantitative PCR (qPCR) analysis of the expression of genes modulated in human cortical spheroids (hCSs) stimulated with IL-1β for 4 h, 12 h or 36 h. Listed are Log2 fold-change (FC) values in the stimulated hCSs over the unstimulated (0 h) hCSs. Correlation RNAseq-qPCR 4 h: 0.758, *p*-value: <0.001; correlation RNAseq-qPCR 12 h: 0.702, *p*-value: 0.001; correlation RNAseq-qPCR 36 h: 0.857, *p*-value: <0.001.
