# Supplementary material for: Molecular Signature of Neuroinflammation Induced in Cytokine-Stimulated Human Cortical Spheroids
Source: Biomedicines. 2022 Apr 29;10(5):1025. doi: 10.3390/biomedicines10051025 (PMC9138619; doi:10.3390/biomedicines10051025)
Supplement: Supplementary file 1 [file biomedicines-10-01025-s001.zip › De Kleijn et al Supplementary figures and tables.pdf]

Supplementary figures and tables

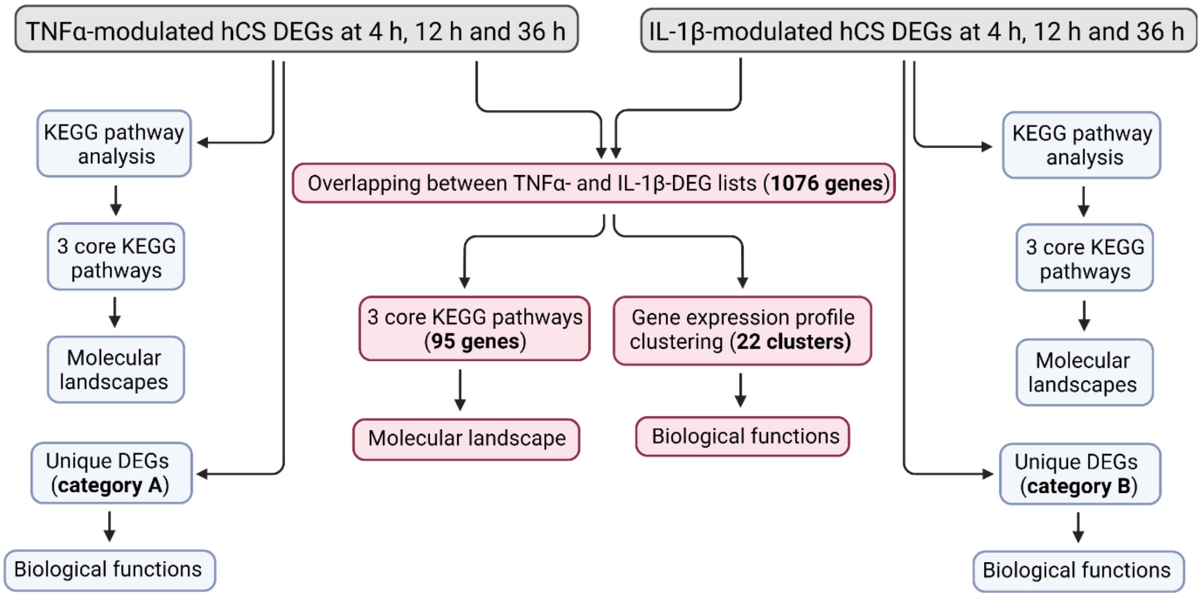

Supplementary Figure S1. Overview of the analysis of differentially expressed genes (DEGs) in human cortical spheroids (hCSs) stimulated with the pro-inflammatory cytokines TNF*α* or IL-1β for 4 h, 12 h and 36 h. Per time point, a molecular landscape for TNF*α*-stimulated or IL-1β-stimulated hCSs was built based on functional interactions between the proteins encoded by the DEGs in the three core annotated KEGG pathways. A further molecular landscape was constructed based on the DEGs that were common in the three core annotated KEGG pathways of the hCSs stimulated with TNF*α* and IL1β for 4 h, 12 h and 36 h. Clustering analysis of the common 1076 DEGs was performed to reveal 22 clusters of time-course gene expression profiles. Also, DEGs unique in TNF*α*- or IL-1β-stimulated hCSs were grouped into a distinct category (category A for TNF*α* and category B for IL-1β). Biological functions were deduced based on information obtained from UniprotKB (https://www.uniprot.org/; 1 January 2022) and Genecards (https://www.genecards.org; 15 January 2022).

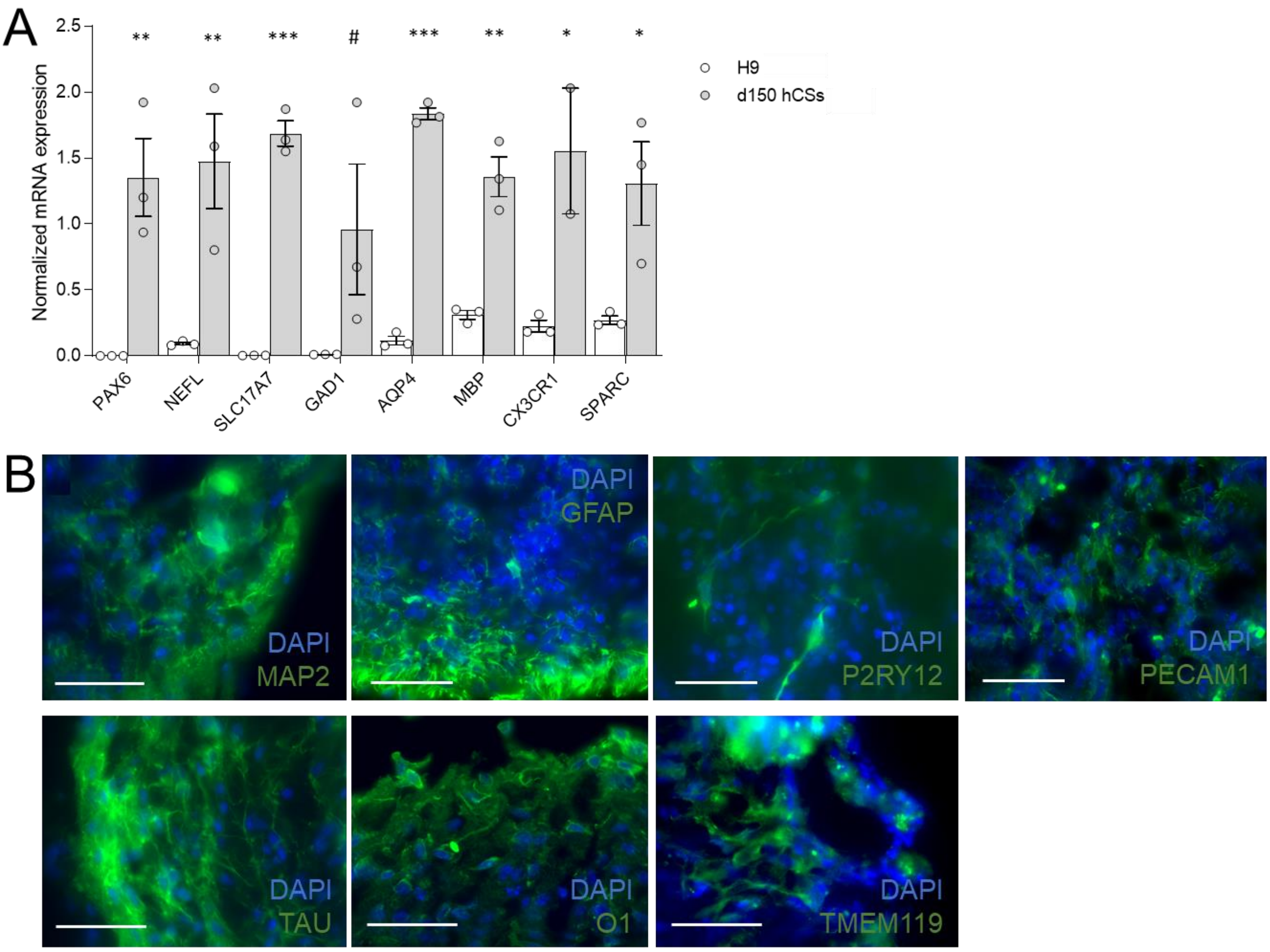

Supplementary Figure S2. Our human cortical spheroids (hCSs) contain neuroectoderm- and mesoderm-derived cell types. **(A)** Levels of mRNA expression (measured by qPCR) of neural progenitor marker *PAX6*, mature neuron marker *NEFL*, excitatory neuron marker *SLC17A7*, inhibitory neuron marker *GAD1*, astrocyte marker *AQP4*, oligodendrocyte marker *MBP*, microglia marker *CX3CR1* and endothelial cell marker *SPARC* in embryonic stem cell line H9 (n=3) and in hCSs (n=3) at day 150 (d150), relative to embryonic H9 stem cells. *** $p < 0.001$, ** $p < 0.01$, * $p < 0.05$, # $p < 0.1$. **(B)** Representative immunocytochemistry images of d150 hCS stainings for neuronal dendritic marker MAP2 and axonal marker TAU, astrocyte marker GFAP, oligodendrocyte marker O1, microglia markers P2RY12 and TMEM119, and endothelial surface marker PECAM1.

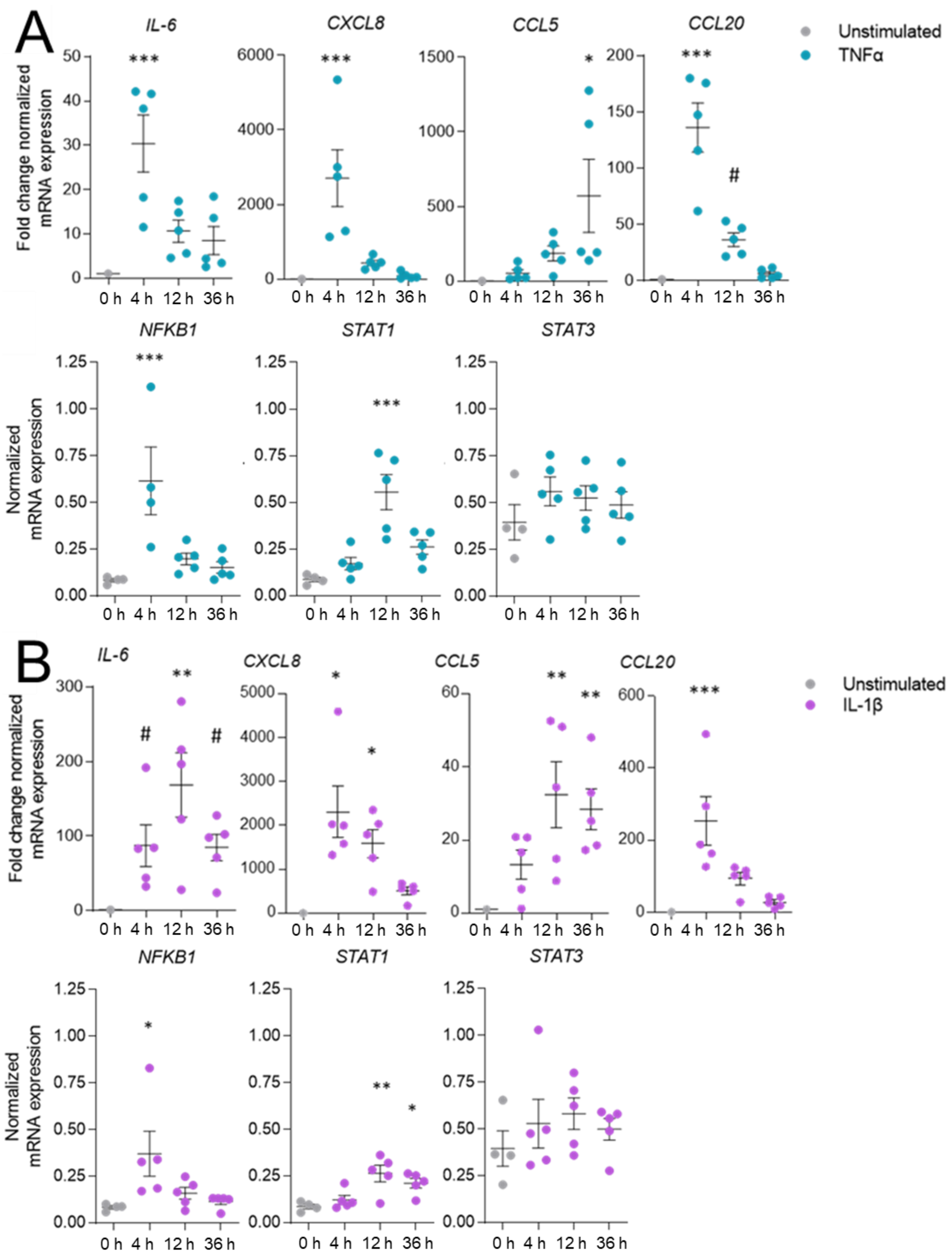

Supplementary Figure S3. Pro-inflammatory stimuli increase mRNA expression of neuroinflammatory-related genes in human cortical spheroids (hCSs). Normalized mRNA expression levels of *IL-6*, *CXCL8*, *CCL5*, *CCL20*, *NFKB1*, *STAT1* and *STAT3* at **(A)** various TNF*α* (5 ng/ml)

incubation time points and **(B)** various IL-1β (5 ng/ml) incubation time points and quantified by qPCR. *** $p < 0.001$, ** $p < 0.01$, * $p < 0.05$, # $p < 0.1$.

A

TNFα-modulated DEGs

4 h

Gene Ontology term	Corrected P-value
Cytokine activity	7.04 × 10−18
Cytokine receptor binding	1.49 × 10−16
Receptor Ligand activity	2.70 × 10−16
Receptor Regulator activity	1.61 × 10−15

12 h

Gene Ontology term	Corrected P-value
Receptor binding	6.26 × 10−14
Cytokine receptor binding	7.52 × 10−12
Glycosaminoglycan binding	8.09 × 10−12
Receptor Ligand activity	8.74 × 10−12

36 h

Gene Ontology term	Corrected P-value
Receptor binding	1.31 × 10−11
Cytokine receptor binding	1.01 × 10−10
Receptor Ligand activity	2.81 × 10−10
Glycosaminoglycan binding	1.94 × 10−09

IL-1β-modulated DEGs

4 h

Gene Ontology term	Corrected P-value
Cytokine activity	4.56 × 10−14
Receptor regulator activity	7.67 × 10−14
Receptor Ligand activity	2.73 × 10−13
Cytokine receptor binding	8.21 × 10−11

12 h

Gene Ontology term	Corrected P-value
Cytokine activity	2.17 × 10−18
Receptor regulator activity	9.18 × 10−17
Receptor Ligand activity	2.03 × 10−16
Cytokine receptor binding	2.21 × 10−14

36 h

Gene Ontology term	Corrected P-value
Receptor binding	4.02 × 10−14
Receptor regulator activity	5.33 × 10−14
Receptor Ligand activity	1.21 × 10−13
Glycosaminoglycan binding	2.80 × 10−10

B

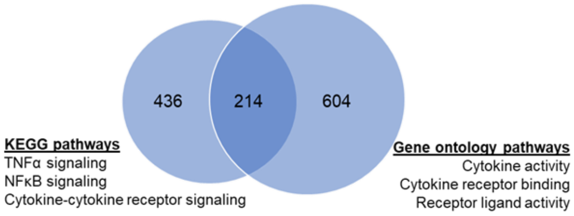

C

TNFα - UP

4 h

Cytokine receptor binding	Corrected P-value
Receptor binding	4.69 × 10−22
Signaling receptor binding	7.95 × 10−19
Cytokine activity	5.3 × 10−18
Receptor ligand activity	2.40 × 10−14

12 h

Cytokine receptor binding	Corrected P-value
Signaling receptor binding	1.19 × 10−17
Cytokine receptor binding	1.12 × 10−16
Cytokine activity	7.45 × 10−14
Binding	1.49 × 10−12

36 h

Cytokine receptor binding	Corrected P-value
Signaling receptor binding	1.54 × 10−17
Cytokine activity	3.18 × 10−12
Binding	3.24 × 10−12
Cytokine receptor binding	3.28 × 10−12

TNFα - DOWN

4 h

Cytokine receptor binding	Corrected P-value
RNA binding	3.00 × 10−04

12 h

Cytokine receptor binding	Corrected P-value
Extracellular matrix structural constituent	3.79 × 10−24
Glycosaminoglycan binding	1.11× 10−10
Heparin binding	1.07× 10−08

36 h

Cytokine receptor binding	Corrected P-value
Extracellular matrix structural constituent	1.74 × 10−12
Nucleic acid binding	1.00× 10−08
RNA binding	4.54× 10−07
Extracellular matrix structural constituent conferring tensile strength	1.13× 10−06

IL-1β - UP

4 h

Cytokine receptor binding	Corrected P-value
Cytokine activity	3.37 × 10−16
Cytokine receptor binding	5.12 × 10−13
Chemokine activity	8.04 × 10−12
Signaling receptor binding	3.37× 10−10

12 h

Cytokine receptor binding	Corrected P-value
Cytokine activity	2.45 × 10−18
Signaling receptor binding	2.52 × 10−18
Cytokine receptor binding	3.57 × 10−18
Binding	1.95× 10−16

36 h

Cytokine receptor binding	Corrected P-value
Signaling receptor binding	2.25 × 10−11
Peptide antigen binding	3.09 × 10−08
Cytokine activity	7.18 × 10−08
Cytokine receptor binding	1.49 × 10−07

IL-1β - DOWN

4 h

Cytokine receptor binding	Corrected P-value
Extracellular matrix structural constituent	5.8 × 10−12
Nucleic acid binding	2.04 × 10−05
RNA binding	2.09 × 10−04
Icosanoid receptor activity	9.95 × 10−04

12 h

Cytokine receptor binding	Corrected P-value
Extracellular matrix structural constituent	5.10 × 10−12
Structural molecule activity	9.65 × 10−04
Glycosaminoglycan binding	9.96 × 10−04

36 h

Cytokine receptor binding	Corrected P-value
Extracellular matrix structural constituent	2.68 × 10−20
Extracellular matrix structural constituent conferring tensile strength	6.16 × 10−08
Glycosaminoglycan binding	6.56 × 10−08
Signaling receptor binding	3.65× 10−07

Supplementary Figure S4. Gene ontology (GO) analysis of differentially expressed genes (DEGs) in human cortical spheroids stimulated with TNFα or IL-1β for 4 h, 12 h and 36 h. **(A)** GO top-4 pathways (based on false discovery rate (FDR)-corrected p-value). **(B)** Number of DEGs in the three core KEGG pathways (left), the three most-often found GO pathways (right) and overlapping between the three core KEGG and GO pathways (214 out of 436 (49.1%) KEGG genes are also GO genes; 214 out of 604 (35.4%) GO genes are also KEGG genes). **(C)** GO top-4 pathways (based on FDR p-value) resulting from the analysis of upregulated DEGs (UP) or downregulated DEGs (DOWN).

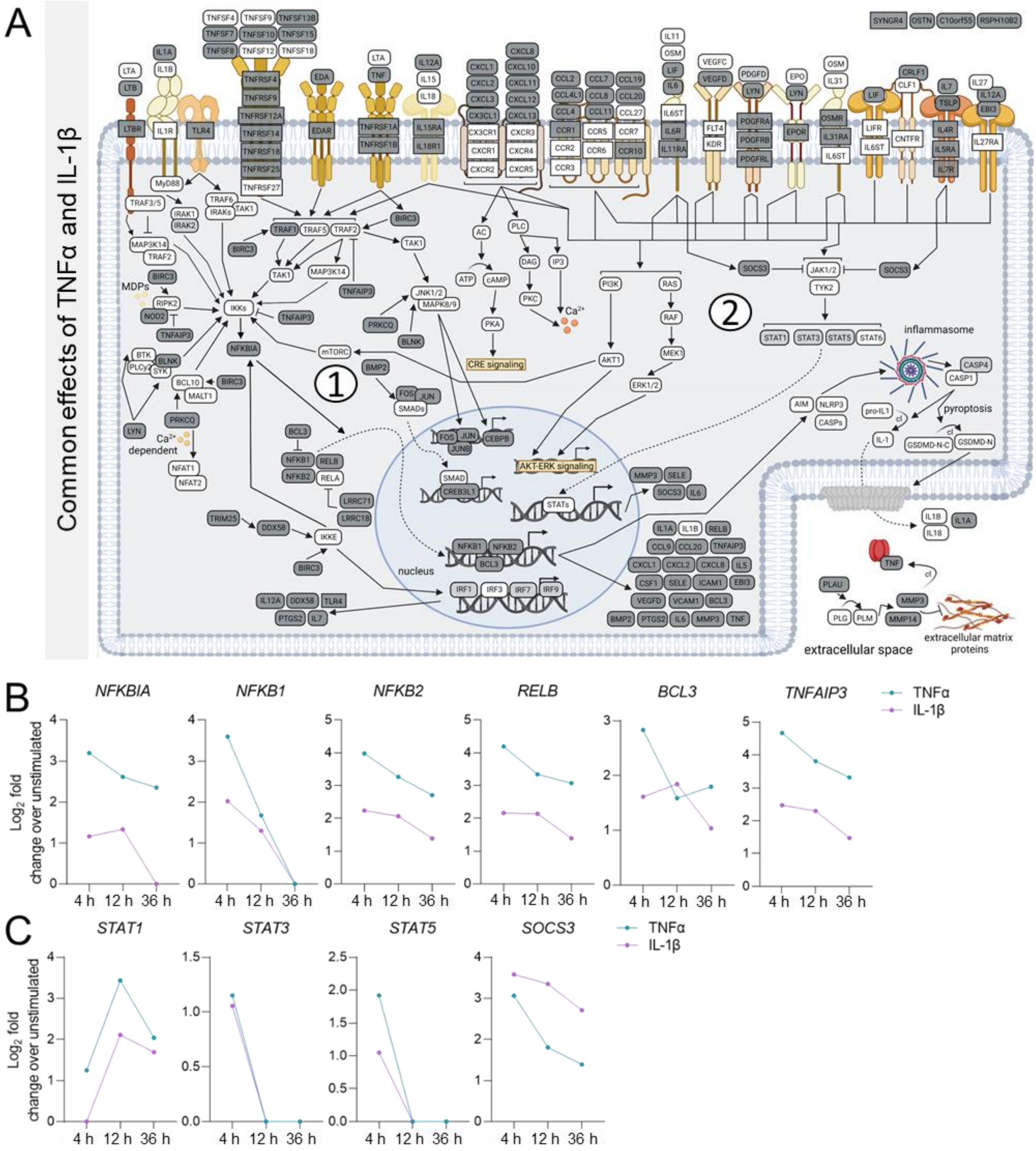

Supplementary Figure S5. (A) Molecular landscape constructed on the basis of differentially expressed genes (DEGs) in human cortical spheroids following stimulation with TNFα and with IL-1β for 4 h, 12 h and 36 h ("common" DEGs). Protein functions and interactions were deduced based on information obtained from UniprotKB (https://www.uniprot.org/; 1 January 2022) and Genecards (https://www.genecards.org; 15 January 2022). A detailed description of the protein-protein interactions occurring in this molecular landscape can be found in Supplementary information 4. Rectangularly framed protein: membrane-bound receptor; protein in dark-gray-filled (rounded) rectangle: encoded by a core KEGG pathway DEG; protein in light-gray-filled (rounded) rectangle: encoded by a DEG not in the core KEGG

pathways; protein in white-filled (rounded) rectangle: not encoded by a DEG. Black arrows: stimulation or induction; inhibition arcs: inhibition; dotted arrows: translocation; fading arrows: enzymatic conversion; cl: cleavage. Encircled numbers refer to the two main dysregulated molecular pathways; 1: NFκB signaling, 2: STAT-dependent transcription. **(B)** Gene expression profiles of NFκB-master regulator genes *NFKBIA, NFKB1, NFKB2, RELB, BCL3* and *TNFAIP3*. **(C)** Gene expression profiles of STAT-master regulator genes *STAT1, STAT3* and *SOCS3.*

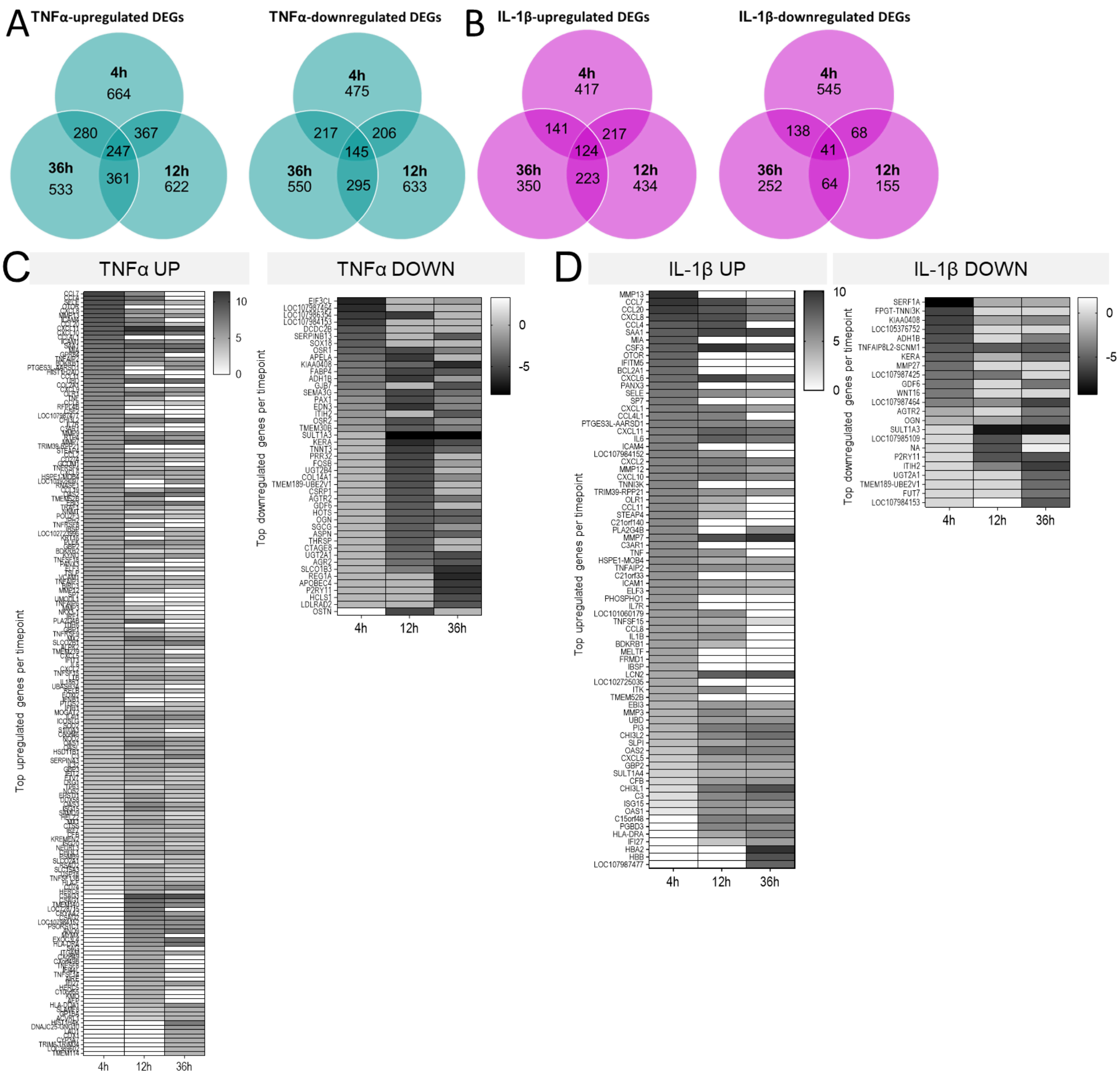

Supplementary Figure S6. Number and time-course expression profiles of upregulated (left) and downregulated (right) differentially expressed genes (DEGs) in human cortical spheroids stimulated with TNFα **(A** and **C)** or IL-1β **(B** and **D)** for 4 h, 12 h and 36 h. For time-course expression profiles, per time point top DEGs are based on Log_2 fold change with a cut-off of 4 (upregulated DEGs; UP) or -4 (downregulated DEGs; DOWN).

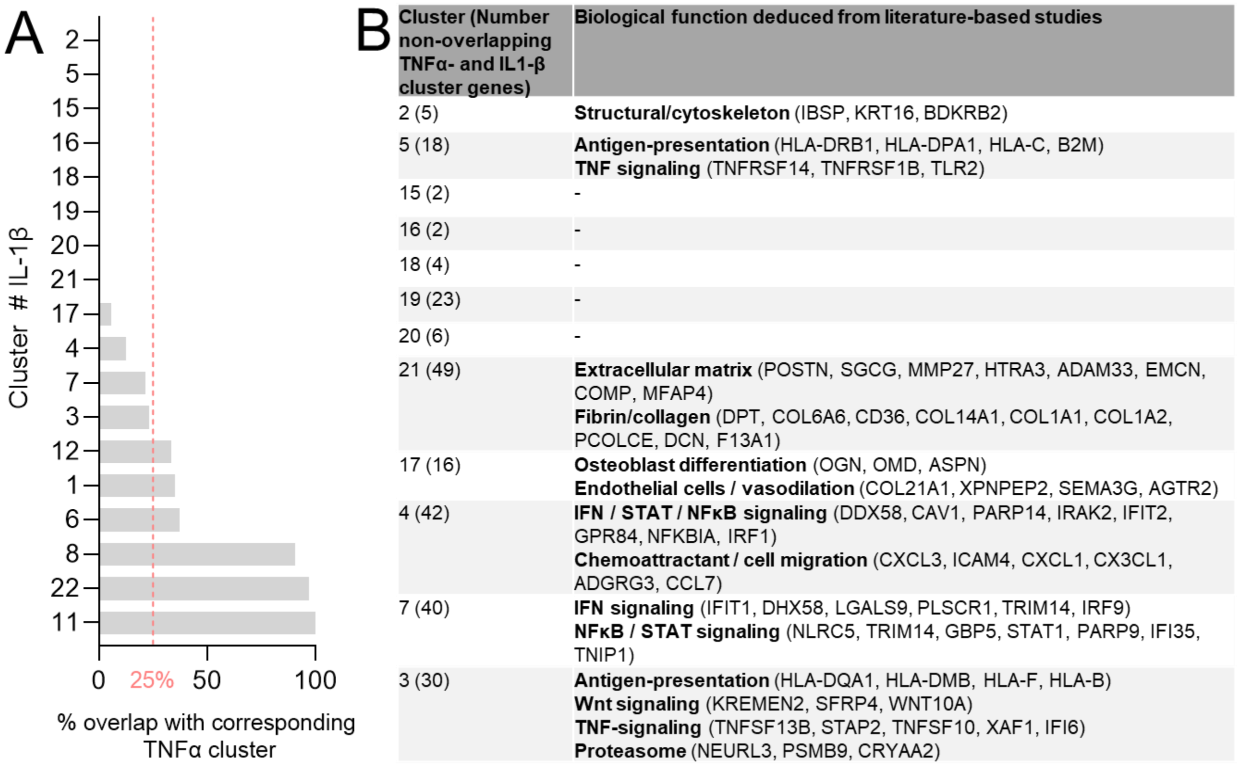

B

Cluster (Number non-overlapping TNFα- and IL1-β cluster genes)	Biological function deduced from literature-based studies
2 (5)	**Structural/cytoskeleton** (IBSP, KRT16, BDKRB2)
5 (18)	**Antigen-presentation** (HLA-DRB1, HLA-DPA1, HLA-C, B2M)<br>**TNF signaling** (TNFRSF14, TNFRSF1B, TLR2)
15 (2)	-
16 (2)	-
18 (4)	-
19 (23)	-
20 (6)	-
21 (49)	**Extracellular matrix** (POSTN, SGCG, MMP27, HTRA3, ADAM33, EMCN, COMP, MFAP4)<br>**Fibrin/collagen** (DPT, COL6A6, CD36, COL14A1, COL1A1, COL1A2, PCOLCE, DCN, F13A1)
17 (16)	**Osteoblast differentiation** (OGN, OMD, ASPN)<br>**Endothelial cells / vasodilation** (COL21A1, XPNPEP2, SEMA3G, AGTR2)
4 (42)	**IFN / STAT / NFκB signaling** (DDX58, CAV1, PARP14, IRAK2, IFIT2, GPR84, NFKBIA, IRF1)<br>**Chemoattractant / cell migration** (CXCL3, ICAM4, CXCL1, CX3CL1, ADGRG3, CCL7)
7 (40)	**IFN signaling** (IFIT1, DHX58, LGALS9, PLSCR1, TRIM14, IRF9)<br>**NFκB / STAT signaling** (NLRC5, TRIM14, GBP5, STAT1, PARP9, IFI35, TNIP1)
3 (30)	**Antigen-presentation** (HLA-DQA1, HLA-DMB, HLA-F, HLA-B)<br>**Wnt signaling** (KREMEN2, SFRP4, WNT10A)<br>**TNF-signaling** (TNFSF13B, STAP2, TNFSF10, XAF1, IFI6)<br>**Proteasome** (NEURL3, PSMB9, CRYAA2)

Supplementary Figure S7. Cluster analysis and biological functions of common differentially expressed genes (DEGs) in human cortical spheroids stimulated with TNFα and IL-1β for 4 h, 12 h or 36 h. **(A)** Percentages of common DEGs in TNFα- and IL-1β-clusters resulting from DEG cluster analysis. Dotted red line: cut-off (overlap score between corresponding TNFα- and IL-1β-clusters < 25%), **(B)** Biological functions of cluster DEGs with a < 25% overlap score between corresponding TNFα- and IL-1β-clusters. Functions were deduced from literature-based studies.

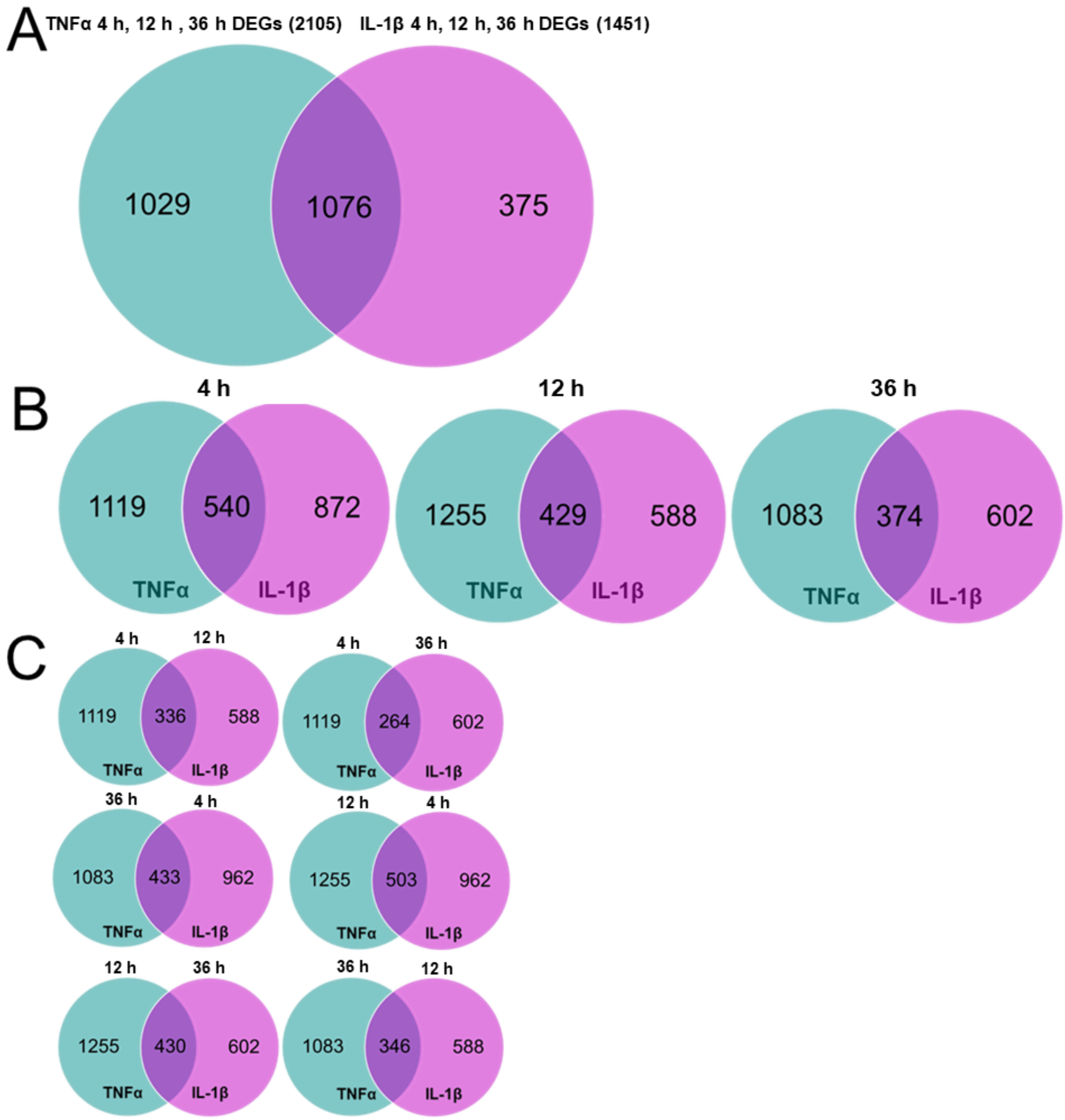

Supplementary Figure S8. Number of differentially expressed genes (DEGs) in human cortical spheroids stimulated with TNFα or IL-1β for 4 h, 12 h and 36 h. **(A)** Number of common DEGs dysregulated at one of the three time-points of TNFα- and IL-1β-stimulation (overlap), and number of DEGs uniquely dysregulated following TNFα-stimulation (left) and IL-1β-stimulation (right). **(B)** Number of common DEGs overlapping between the TNFα- and IL-1β-DEG lists (overlap), and number of DEGs unique to the TNFα-DEG list (left) and IL-1β-DEG list (right) per stimulation time point. **(C)** Number of common DEGs overlapping between the TNFα- and IL-1β-DEG lists (overlap), and number of DEGs unique to the TNFα-DEG lists (left) and IL-1β-DEG lists (right) among the various stimulation time points.

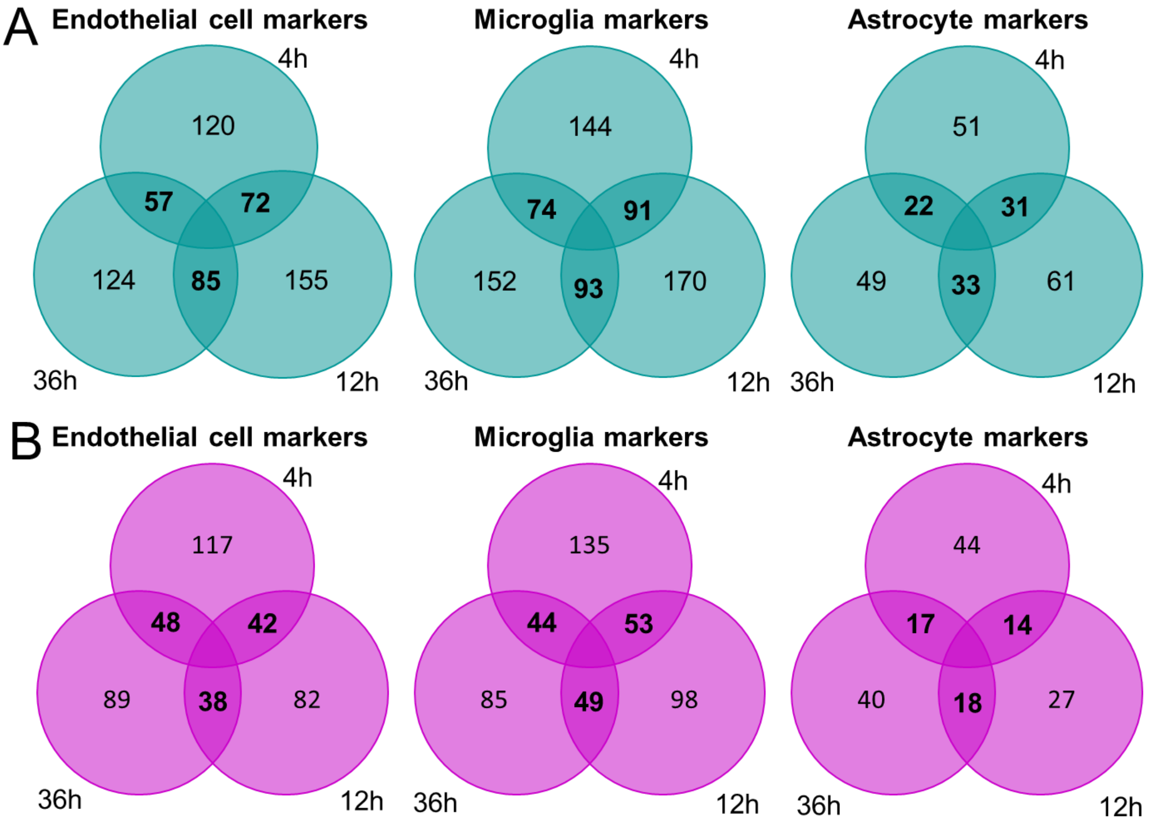

Supplementary Figure S9. Number of endothelial cell-, microglia- and astrocyte-marker genes that are dysregulated in human cortical spheroids following stimulation with **(A)** TNFα or **(B)** IL-1β for 4 h, 12 h or 36 h. Marker genes (Supplementary information 3) were based on a single-cell RNA-seq dataset of human fetal brain regions [25].

Supplementary Table S1. Boolean clustering of the time-course expression profiles of common differentially expressed genes (DEGs) selected on the basis of their presence in the DEG-lists of human cortical spheroids (hCSs) stimulated with TNFα for 4 h, 12 h or 36 h as well as in the DEG-lists of hCSs stimulated with IL-1β for 4 h, 12 h or 36 h. In the column "Pattern", "high" refers to a Log_2 fold change (FC) > 2.5 or < -2.5 and "low" refers to a Log_2 FC between 0 and 2.5 or between -2.5 and 0. In the column "Direction", "pos" indicates that the FC-value is above 0 and "neg" indicates that the FC-value is below 0. The boundary at the DEG threshold of Log_2 FC = 1 could not be set as some genes were only a DEG at one time point and thus values with a Log_2 FC between 1 and -1 were also present in the dataset.

**Cluster #**	**4 h**	**12 h**	**36 h**	**Pattern**	**Direction**
1	> 2.5	>2.5	> 2.5	high high high	pos pos pos
2	> 2.5	> 0, < 2.5	> 2.5	high low high	pos pos pos
3	> 0, < 2.5	> 2.5	> 2.5	low high high	pos pos pos
4	> 2.5	> 2.5	> 0, < 2.5	high high low	pos pos pos
5	> 0, < 2.5	> 0, < 2.5	> 2.5	low low high	pos pos pos
6	> 2.5	> 0, < 2.5	> 0, < 2.5	high low low	pos pos pos
7	> 0, < 2.5	> 2.5	> 0, < 2.5	low high low	pos pos pos
8	> 0, < 2.5	> 0, < 2.5	> 0, < 2.5	low low low	pos pos pos
9	> 0	< 0	> 0		pos neg pos
10	< 0	> 0	< 0		neg pos neg
11	> 0	> 0	< 0		pos pos neg
12	> 0	< 0	< 0		pos neg neg
13	< 0	> 0	> 0		neg pos pos
14	< 0	< 0	> 0		neg neg pos
15	<-2.5	<-2.5	<-2.5	high high high	neg neg neg
16	<-2.5	<0, > -2.5	<-2.5	high low high	neg neg neg
17	<0, > -2.5	<-2.5	<-2.5	low high high	neg neg neg
18	<-2.5	<-2.5	<0, > -2.5	high high low	neg neg neg
19	<0, >-2.5	<0, > -2.5	<-2.5	low low high	neg neg neg
20	<-2.5	<0, > -2.5	<0, > -2.5	high low low	neg neg neg
21	<0, > -2.5	<-2.5	<0, > -2.5	low high low	neg neg neg
22	<0, > -2.5	<0, > -2.5	<0, > -2.5	low low low	neg neg neg

Supplementary Table S2. Listed are per cluster (see Figure 4) the percentages of TNFα-modulated differentially expressed genes (DEGs) and the percentages of IL-1β-modulated DEGs. Percentages are relative to the total number of common TNFα- and IL-1β-modulated DEGs (1076 DEGs).

**Cluster** #	**% TNFα modulated DEGs**	**% IL-1β modulated DEGs**
1	6.88	3.53
2	0.46	0.19
3	3.62	2.51
4	4.46	2.51
5	1.67	0.74
6	5.48	3.90
7	4.74	3.16
8	22.68	32.90
9	0	0
10	0	0
11	0.09	0.19
12	0.28	0.19
13	0	0.09
14	0	0
15	0.19	0.19
16	0.19	1.11
17	1.56	0.46
18	0.37	0.09
19	2.14	1.67
20	0.56	0.74
21	4.55	0.19
22	39.13	45.17

Supplementary Table S3. Listed are per cluster (see Figure 4) the number of genes in the time-course expression cluster of TNFα- and IL-1β-modulated differentially expressed genes (DEGs), the number of genes common in the time-course expression clusters of TNFα- and IL-1β-modulated DEGs and the percentage of genes common in the time-course expression clusters of TNFα- and IL-1β-modulated DEGs.

**Cluster #**	**# genes in TNFα-cluster**	**# genes in IL-1β-cluster**	**# genes common in TNFa- and IL-1β clusters**	**% genes common in TNFa- and IL-1β clusters (% relative to number of TNFα-cluster genes)**
1	74	38	26	35.1
2	5	2	0	0
3	39	27	9	23.1
4	48	27	6	12.5
5	18	8	0	0
6	59	42	22	37.3
7	51	34	11	21.6
8	244	354	221	90.6
9	0	0	-	-
10	0	0	-	-
11	1	2	1	100
12	3	2	1	33.3
13	0	1	0	0
14	0	0	-	-
15	2	2	0	0
16	2	12	0	0
17	17	5	1	5.9
18	4	1	0	0
19	23	18	0	0
20	6	8	0	0
21	49	2	0	0
22	421	486	408	96.9

Supplementary Table S4. Primer sequences used for qPCR validation of RNA-seq data in day-150 human cortical spheroids.

Transcript	Forward sequence	Reverse sequence
AQP4	GGAAATTGGGAAAACCATTGGA	AAGACATACTCATAAAGGCCACCA
CCL20	GCTGTGACATCAATGCTATCATCT	CTTTTTACTGAGGAGACGCACAA
CCL5	CCATATTCCTCGGACACCACAC	CGAACCCATTTCTTCTCTGGGT
CX3CR1	CAAATGCCACACATCACCCTTA	TTTGACTCGATGCAGTAGGCAG
CXCL8	GATTTCTGCAGCTCTGTGTGAAGG	GGGTGGAAAGGTTTGGAGTATGT
GAD1	CCATTCTCGTCAAGGAAAAGGG	ACTGCTTGTCTGGCTGGAAGAG
GAPDH	GTCATGGGTGTGAACCATGAGA	GCATGGACTGTGGTCATGAGTC
GFAP	CAGGACCTGCTCAATGTCAAGC	GTTTCTCGAATCTGCAGGTTGG
IL-6	TGAGGAGACTTGCCTGGTGAA	ACTCATCTGCACAGCTCTGGCT
MBP	ACCCAAGATGAAAACCCCGTA	GAAATGGCTCGTCACCTTCGT
NEFL	GACCCTGGAAATCGAAGCATG	TTGATCGTGTCCTGCATAGCG
NFKB1	TGATCTGTACCAGACGCCCTTG	TCTTTGGCAGCTAGGTGCAAA
PAX6	CAAATAACCTGCCTATGCAACCC	GCTGACTGTTCATGTGTGTCTGC
SLC17A7	GTTGATGAACTGCGGAGGCTTC	GTCCAGGTGGTTCACGTTGAAC
SPARC	TACATCGGGCCTTGCAAATAC	TGTCCTCATCCCTCTCATACAGG
STAT1	TCGACAGTCTTGGCACCTAAC	GTACCACTGAGACATCCTGCC
STAT3	ACCATTGACCTGCCGATGTC	AAGGTGAGGGACTCAAACTGC
YWHAZ	CGCTGGTGATGACAAGAAAGG	GAAGTTAAGGGCCAGACCCAGT

Supplementary Table S5. Validation of RNA-sequencing (RNA-seq) data by quantitative PCR (qPCR) analysis of the expression of genes modulated in human cortical spheroids (hCSs) stimulated with TNFα for 4 h, 12 h or 36 h. Listed are Log_2 fold-change (FC) values in the stimulated hCSs over the unstimulated (0 h) hCSs. Correlation RNAseq-qPCR 4h: 0.685, p-value : 0.001; correlation RNAseq-qPCR 12h: 0.862, p-value : < 0.001; correlation RNAseq-qPCR 36h: 0.819, p-value : < 0.001.

Gene	Log_2 FC 4 h over 0 h (RNA-seq)	Log_2 FC 12 h over 0 h (RNA-seq)	Log_2 FC 36 h over 0 h (RNA-seq)	Log_2 FC 4 h over 0 h (qPCR)	Log_2 FC 12 h over 0 h (qPCR)	Log_2 FC 36 h over 0 h (qPCR)
CASP1	-0.41328	0.473297	0.701659	-0.22733	0.622238	0.391013
NFKB1	3.595265	1.673808	0.846103	3.058115	1.248208	0.864795
IL1B	0	-0.58496	-1.58496	-0.70152	0.415028	0.9347
GSDMD	-0.47846	0.71114	1.317267	-0.45102	-0.36782	0.448086
IL-6	4.299208	3.055282	1.459432	4.923394	3.408947	3.082219
TNF	6.285402	4.807355	3.906891	8.246019	6.349774	6.304054
CASP8a	-0.31015	-0.22476	-0.10292	-0.52994	-0.36844	-0.17874
CASP3	0.392343	0.077863	0.147918	0.373353	0.249784	0.165412
NEFL	0.261588	-0.32883	0.07761	0.161389	-0.62918	-0.0046
PLP1	-0.12062	-0.63304	-0.71647	-0.18907	-0.67546	-0.78794
GFAP	0.767701	1.801595	1.197088	0.812531	1.424179	0.650184
FGFR3	0.318682	0.203747	0.606416	-0.12235	0.092705	0.256252
CXCL8	9.211348	6.366322	3.345775	11.40253	8.762586	6.51757
S100B	0.023744	0.025109	-0.18405	0.243255	0.41676	-0.20017
TGFB2	-0.67413	-0.4967	-0.37367	-0.44422	-0.60363	-0.31222
CCL5	0.36728	1.83064	3.031784	5.747325	7.540873	9.158498
CCL20	8.73471	6.514573	3.30117	7.089191	5.186672	2.634049
MAP2	-0.14105	0.061004	0.156159	-0.04027	0.11295	0.127895

Supplementary Table S6. Validation of RNA-sequencing (RNA-seq) data by quantitative PCR (qPCR) analysis of the expression of genes modulated in human cortical spheroids (hCSs) stimulated with IL-1β for 4h, 12h or 36h. Listed are Log_2 fold-change (FC) values in the stimulated hCSs over the unstimulated (0 h) hCSs. Correlation RNAseq-qPCR 4h: 0.758, p-value : < 0.001; correlation RNAseq-qPCR 12h: 0.702, p-value : 0.001; correlation RNAseq-qPCR 36h: 0.857, p-value : < 0.001.

Gene	Log_2 FC 4 h over 0 h (RNA-seq)	Log_2 FC 12 h over 0 h (RNA-seq)	Log_2 FC 36 h over 0 h (RNA-seq)	Log_2 FC 4 h over 0 h (qPCR)	Log_2 FC 12 h over 0 h (qPCR)	Log_2 FC 36 h over 0 h (qPCR)
CASP1	-1.77083	0.325095	-0.89308	-1.16012	-0.12961	-0.7904
NFKB1	2.018132	1.298876	0.421107	2.154579	0.93137	0.460119
IL1B	4.662965	5.142958	2.736966	1.594562	1.289886	0.974264
GSDMD	-1.09622	0.255257	-0.18601	-0.6714	-0.09302	-0.65311
IL-6	6.186114	7.806147	6.622052	6.445521	7.398288	6.403687
TNF	5.584963	5.169925	3.70044	7.024378	6.061383	4.369505
CASP8a	-0.51796	0.290366	0.198546	-0.1551	0.181148	-0.01531
CASP3	0.596747	-0.093	0.058661	0.63501	0.211932	0.031096
NEFL	-0.27673	-0.03538	0.295237	-0.12107	-0.20689	0.040685
PLP1	0.204718	-0.51565	-0.73194	0.223242	-0.38046	-0.72151
GFAP	1.264698	1.695778	2.200887	1.04312	1.276357	1.821861
FGFR3	1.382248	-0.10941	0.738586	0.587332	-0.39051	0.228926
CXCL8	8.895491	8.586423	6.674781	11.16951	10.62913	8.981241
S100B	0.185035	0.274382	0.69857	0.427569	0.372366	0.614885
TGFB2	-0.60098	-0.33871	0.096802	-0.30158	-0.23024	0.06799
CCL5	0.356267	0.331488	-0.29178	3.725013	5.016357	4.827882
CCL20	9.485829	8.194757	6.042832	7.984097	6.559726	4.812155
MAP2	-0.10021	-0.09997	0.224675	-0.02979	0.066822	0.098418
