# Supplementary material for: Molecular Signature of Neuroinflammation Induced in Cytokine-Stimulated Human Cortical Spheroids
Source: Biomedicines. 2022 Apr 29;10(5):1025. doi: 10.3390/biomedicines10051025 (PMC9138619; doi:10.3390/biomedicines10051025)
Supplement: Supplementary file 1 [file biomedicines-10-01025-s001.zip › Supplementary information 4.pdf]

Supplementary information 4 - refers to Supplementary Figure S5A

LTBR is a receptor for LTA and LTB, and signals via TRAF3 and TRAF5 to inhibit TRAF2-MAP3K14-mediated activation of NF-kappa-B (NFκB) via proteasomal degradation of MAP3K14, reducing inhibitory-κB kinase (*IKK/IKBK*)-dependent post-translational protein modification (phosphorylation) of NFκB inhibitor-alpha (NFKBIA) [82-84]; the IKBK-complex is the core element of the NFκB cascade. IL1R serves as a receptor for IL1A and IL1B, and activates the IKBK subfamily via the signaling adapter molecules MyD88, IRAK1 and IRAK2 that induce IKBK-dependent phosphorylation of NFKBIA [85-88]. TLR4 activates the IKBK subfamily and subsequent proteolytic processing of NFKBIA via the signaling adapter molecule MyD88 or via IRAK-TRAF6-TIK1-signaling [89, 90]. TNFRSF4, TNFRSF9, TNFRSF12A, TNFRSF14, TNFRSF18, TNFRSF25 and TNFRSF27 are TNF receptors for the ligands TNFSF4, TNFSF7, TNFSF8, TNFSF9, TNFSF10, TNFSF12, TNFSF13B, TNFSF15 and TNFSF18, and activate proteolytic processing of NFκB-subunits to allow their translocation to the nucleus via TRAF1-IKBK-NFKBIA, TRAF2-IKBK-NFKBIA, TRAF5-IKBK-NFKBIA and TRAF2-MAP3K14 signaling [91-99]. EDAR is a receptor for EDA that signals through TRAF2-IKBK-NFKBIA and activates c-JUN N-terminal kinase (*JNK*) signaling [100]. TNF and LTA (in its homotrimeric form) bind to both TNFRSF1A (alias TNFR1) and TNFRSF1B (alias TNFR2), and as such activate TRAF1-TRAF2-IKBK-NFKBIA signaling [101]. TNFAIP3 is a direct inhibitor of IKBK-family members, and inhibits IKBKs also via inhibition of TRAF2 and of the NOD2-RIPK2 complex that forms in response to muramyl dipeptide (MDP) recognition [102]. BLNK activates IKBK-dependent NFKBIA phosphorylation via the BLNK-SYK-BTK-PLCγ2 complex [103] and activates the JNK pathway [104]. The inflammation-transcription factor CEBPB is activated via mitogen-activated protein k*inase (MAPK) kinases* (*MKKs*) that include JNK1/2 [105]. LYN phosphorylates both BTK and SYK [106]. The expression of BCL3 is also induced by NFκB, which is part of the self-regulatory feedback loop that inhibits the translocation of NFKB1 from the cytoplasm to the nucleus [107], and BCL3 acts in the nucleus as a transcriptional coactivator of NFκB-subunits following phosphorylation by IKBKs [108, 109]. PRKCQ mediates translocation and activation of NFKB1 and NFKB2 through activation of the BCL10-MALT1 complex and IKBK [110], and activates the transcription factor JUN via JNK signaling [111, 112]. BIRC3 is an E3 ubiquitin-protein ligase for RIPK1/2/3/4, IKBKE, TRAF1 and BCL10, which results in downstream IKBK-dependent NFKBIA phosphorylation [113, 114]. NFκB-subunits NFKB1, NFKB2, RELA and RELB are disinhibited following phosphorylation of NFKBIA and translocate to the nucleus where the homo- or heterodimeric complexes bind to DNA to activate or repress transcription [115, 116]. LRRC71 and LRRC18 may inhibit RELA, as one of their family members (LRRC25) displays a similar function [117]. NFκB binding to DNA regulates transcriptional activation of CSF1 [118], SELE [119, 120], IL6 [121, 122], ICAM1 and VCAM1 [123, 124], PTGS2 [125, 126], VEGFD [127], CXCL1 [128], CXCL2 [128], IL11 [129], CXCL8/IL8 [130, 131], IL1B [132], IL1A [133], EBI3 [134], CCL9 [135], CCL20 [136, 137], TNF [138, 139], TNFRSF9 [140], BMP2 [141, 142], TNFAIP3 [143], IRF7 [144], RELB [145] and the extracellular matrix metalloproteinase MMP3 [146, 147]. MMP3 and MMP14 degrade extracellular matrix proteins such as fibronectin, laminin and collagens [148, 149] and MMP3 cleaves a subdomain of TNFα [150]. Plasminogen activator urokinase (PLAU) converts PLG to PLM, and PLM cleaves pro-MMPs to active MMPs [151, 152].

CCR1, CCR2, CCR3, CCR5, CCR6, CCR7 and CCR10 are receptors for the cytokines CCL2, CCL4, CCL4L1, CCL7, CCL8, CCL11, CCL19 and CCL20, and activate the JAK-STAT-signaling pathway [153], MAPK-signaling pathway and PI3K-AKT1-signaling via JNK [154-159]. The chemokines CX3CL1, CXCL1, CXCL2, CXCL3, CXCL8, CXCL10, CXCL11, CXCL12 and CXCL13 bind to CX3CR1, CXCR1, CXCR2, CXCR3, CXCR4 and CXCR5, and induce GPCR-signaling via PI3K-AKT1-, PLC/DAG- and MAPK-signaling [160-164].

IL6R associates with IL6ST to form a receptor for IL6, LIF and OSM (among others) [165] and activates MEK1-ERK1/2-signaling [166] and STAT3-signaling [167]. IL6-signaling also activates the JAK-repressor SOCS3 [168]. IL4R, IL5R, IL7R, via their ligands IL4/IL13,

IL5 and IL7/TSLP, respectively, activate JAK-STAT signaling [38, 169-171]. A complex consisting of IL12A, IL27 and EBI3 binds to IL31RA to activate JAK-STAT-signaling [172-174] and SOCS3 [175, 176]. IL15R and IL18R1 are receptors for IL12A, IL15 and IL18, and activate JAK-STAT-signaling and TRAF2/6-IKBK-dependent NFκB signaling [177, 178]. VEGFD is a ligand for KDR and FLT4, tyrosine kinase receptors that activate RAS-RAF-MEK1-ERK1/2- and PI3K-AKT1-signaling [179, 180]. VEGFD also promotes nuclear translocation of STAT1/STAT6 via KDR/FLT4 [181]. PDGFRA, PDGFRB and PDGFRL activate ERK1/2- and AKT1-signaling [182, 183], and JAK1-STAT1/3/6- and JAK2-STAT5 signaling [184, 185] through activation by PDGFB, PDGFD or LYN [186]. Upon binding of EPO and LYN, EPOR activates the PI3K-AKT1-, ERK1/2- and JAK2-STAT5-signaling pathways [106, 187]. OSMR in complex with IL6ST binds to IL31RA to form IL31R, and activate STAT1, STAT3 and STAT5 via JAK2 [188, 189]. Upon binding of LIF, LIFR in complex with IL6ST activates JAK-STAT-signaling [190, 191], highly similar to the OSM-OSMR mechanism of action [192]. Likewise, a complex between IL11RA and IL6ST activates JAK-STAT- and PI3K-AKT1-signaling [193]. CRLF1 and CLCF1 bind to CNTFR in complex with LIFR and IL6ST to activate JAK-STAT- and ERK1/2-signaling [194-197]. AKT1-mTOR-signaling activates IKBK-dependent phosphorylation of NFKBIA [198-200] and the NFκB pathway via mechanisms independent of NFKBIA degradation [201]. Increased STAT-signaling results in MMP3, SELE, SOCS3 and IL6 transcription [168, 202, 203]. Downstream of JNK1/2 signaling, the nuclear heterodimeric transcription factor complex activator protein *1* (AP-1; predominant form consisting of FOS and JUN family members) regulates (among other genes) inflammatory gene expression [204]. FOS and JUN also form a multimeric complex with SMAD3 and SMAD4 to regulate TGF-β signaling [205]. BMP2 activates SMAD1/5/8 and SMAD signaling via BMPR2 [206]. CREB3L1 binds to SMAD4 regulating collagen-containing extracellular matrix genes [207], is a transcription factor controlling the cell-cycle following viral infection and is produced via p38-MAPK or the nuclear receptor NR4A1 [208].

DDX58 is polyubiquinated and activated by TRIM25 [209, 210], and activates IKBKE which results in phosphorylation of IRF3 and IRF7, inducing interferon signaling [211, 212]. Increased transcription of PTGS2 [213], IL7 [214], DDX58 [215], IL12A [216] and TLR4 [217] is a result of IRF3/7-dependent interferon signaling. IL1A, IL1B and IL18 (cleaved by CASPs from pro-IL1A, pro-IL1B and pro-IL18, respectively) are secreted following the formation of GSDMD-cleavage-induced membrane pores upon NLRP3-inflammasome activation and nuclear translocation of NFκB-subunits.

In flagella and motile cilia, RSPH10B2 is a component of the radial spoke head of which the mechanism of action is unclear [218].

To our knowledge, no protein-protein interactions that fit into this landscape have been reported for the proteins encoded by the differentially expressed genes SYNGR4, OSTN and C10orf55.
